# Homogeneously‐Dimensionalizing Perovskite Surface by Dual‐Mechano‐Chemical Regulation for Efficient Solar Cells

**DOI:** 10.1002/advs.202509089

**Published:** 2025-07-13

**Authors:** Shengwei Geng, Jialong Duan, Chenlong Zhang, Jinyue Zhang, Yueyang Bi, Yueji Liu, Xixi Zhu, Xinyu Zhang, Qiyao Guo, Jie Dou, Benlin He, Yuanyuan Zhao, Qunwei Tang

**Affiliations:** ^1^ College of Chemical and Biological Engineering Shandong University of Science and Technology Qingdao 266590 P. R. China; ^2^ Wuhan National Laboratory for Optoelectronics and School of Physics Huazhong University of Science and Technology Wuhan 430074 P. R. China; ^3^ School of Materials Science and Engineering Ocean University of China Qingdao 266100 P. R. China; ^4^ College of Energy Storage Technology Shandong University of Science and Technology Qingdao 266590 P. R. China

**Keywords:** charge transfer, dimensionality heterointerface, perovskite solar cells, stability, surface polishing

## Abstract

Precise manipulation on surface dimensionality benefits the improvement of efficiency and stability of perovskite solar cells, however, heterogeneity with the presence of substantial atomic‐scale impurities and micro‐wrinkles on perovskite surface that serve as transformation template challenges the formation of homogeneous heterointerface and thus weakens healing efficacy. To address this issue, herein, we propose a dual‐mechano‐chemical strategy is proposed to homogenize the morphologic‐compositional feature of perovskite surface by first polishing superficial nano‐impurities with energetic nanoparticles and then in situ dimensionalizing the defect‐free lattice to form a 2D/3D heterointerface with strengthened contact and homogeneous distribution. With the implement of this strategy, the reconstructed heterointerface not only accelerates charge transfer with minimized interfacial non‐radiative recombination losses, but also protects perovskite lattice from external attack. Consequently, an all‐air‐processed carbon‐based CsPbI_2_Br solar cell displays enhanced efficiency of 15.29% and elevated performance retention rate under dark storage over 1000 h, high temperature over 500 h as well as persistent operation over 200 h. This work provides a multidimensional surface engineering strategy for high‐efficiency and stable perovskite‐based photoelectric device, benefiting the large‐scale fabrication in the future.

## Graphical abstract

A homogeneous and strengthened 2D/3D perovskite heterointerface is realized by idealizing the perovskite surface to eliminate atomic‐scale impurities and micro‐wrinkles. Benefiting from the reinforcement of charge transfer and lattice solidification, an all‐air‐processed carbon‐based all‐inorganic CsPbI_2_Br device achieves an enhanced efficiency of 15.29% with improved stability, offering a deep insight on dimensionality engineering.

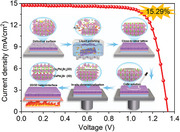

## 1 Introduction

With the certified power conversion efficiency (PCE) up to 27.0%, perovskite solar cells (PSCs) have shown great competitiveness to the commercial photovoltaic market, as they combine the merits of easy fabrication and engineering with respect to film, and low manufacturing cost for the final module.^[^ ^1^, ^2^, ^3^, ^4^, ^5^ ^]^ Till now, in addition to the efficiency that has already meet the fundamental requirement for large‐scale photovoltaic industry, another noticeable concern on the device fatigue behavior under external stressors (such as moisture, heat, oxygen and light) that is a crucial factor in determining the practical application, still poses a major limitation for further development of PSC from laboratory to factory, which calls for continual excitements into the technological update for elevating the durability.^[^ ^6^, ^7^, ^8^, ^9^ ^]^ In general, the predominant origin of the instability for PSCs is closely associated with the presence of multi‐defects in polycrystalline perovskite film. Compared to bulk interior, the exposed vulnerable surface always shows more atomic‐scale impurities and micro‐wrinkles owing to the uneven crystallization dynamics and preferred moisture/oxygen erosion.^[^ ^10^, ^11^, ^12^ ^]^ In detail, variations in surface tension caused by solvent evaporation trigger challenging solvent flows, such as Marangoni convection and capillary flow, which commonly cause uneven solute distribution, manifesting as the coffee ring effect and orange peel effect.^[^ ^13^ ^]^ Furthermore, as for the mixed‐halide wide‐bandgap perovskite film, local halide heterogeneity is another driven force for the formation of surface wrinkling, mainly attributed to the difference of crystallization rate between Br‐rich and I‐rich phases. The faster crystallization of Br‐rich phase forms a skin layer with a higher elastic modulus than the wet film below. As the I‐rich film gradually crystallizes and shrinks, a compressive stress on the top layer is produced, leading to the formation of the wrinkling.^[^ ^10^ ^]^ Consequently, a soft superficial layer with defective lattice, morphologic‐compositional heterogeneity, and residual strain contributes to the structural collapse of the perovskite lattice, photogenerated carrier recombination, and photovoltaic performance degradation. So far, various surface polishing techniques have been proposed to remove this defective layer, such as using high‐energy lasers, adhesive tape, and ChemoMet soft pad.^[^ ^14^, ^15^, ^16^ ^]^ With the implementation of these methods, the efficiency and stability of PSC have been significantly improved. In this fashion, developing strategies to effectively heal perovskite surface (or remove the wrinkling) and improve fatigue behavior is important for obtaining applicable PSCs.

Actually, great achievements on stability improvement have been obtained with the popular use of diverse organics, such as Lewis bases for passivating halide vacancy defects owing to its lowest formation energy,^[^ ^17^, ^18^ ^]^ ammonium salts for constructing two‐dimensional (2D) capping layer,^[^ ^19^ ^]^ and even polar molecules for polishing the defective layer.^[^ ^20^, ^21^ ^]^ Among them, the formation of 2D/3D heterointerface has demonstrated superior resistance to moisture and oxygen, that helps improve the film stability.^[^ ^22^ ^]^ However, based on the above discussions, the heterogeneity in defect distribution and morphological variation on the perovskite surface that serves as a growth template for 2D phase inevitably induces the formation of inhomogeneous and weakened heterointerface, which adversely maximizes the healing efficacy of low‐dimension capping layer.^[^ ^23^, ^24^ ^]^ In addition, recent studies have demonstrated that these organic molecules suffer from irreversible photothermal decomposition and even uncontrollable insertion into perovskite bulk during real‐world operation conditions owing to the low binding energies of these phases^[^ ^25^, ^26^, ^27^ ^]^ leading to the performance deterioration.

In avoiding this contradictory effect, in this work, we propose a dual‐mechano‐chemical strategy to effectively dimensionalize perovskite surface by first utilizing energetic Al_2_O_3_ nanoparticles as a polishing agent and then all‐inorganic 2D Cs_2_PbI_2_Br_2_ perovskite as a solidified layer. Benefitting from the removal of superficial nano‐impurities and morphologic micro‐peaks, the exposed surface with nearly close‐to‐ideal lattice homogenizes the formation of 2D perovskite capping layer, which in turn strengthens the 2D/3D heterointerface for simultaneously minimizing charge recombination behavior and withstanding external stimuli. After optimization, we finally obtain an all‐air‐processed carbon‐based CsPbI_2_Br solar cell with an efficiency of 15.29% by utilizing this synergetic surface reconstruction strategy, one cutting‐edge value among the congeneric cells. More importantly, the champion device exhibits excellent stability (elevated performance retention) under dark storage over 1000 h, high temperature over 500 h, and persistent operation over 200 h. Considering the universal challenge for other perovskites, our findings provide a feasible approach to idealize the defective surface for the commercialization of perovskite‐based optoelectronics.

## 2 Results and Discussion

**Figure 1:**
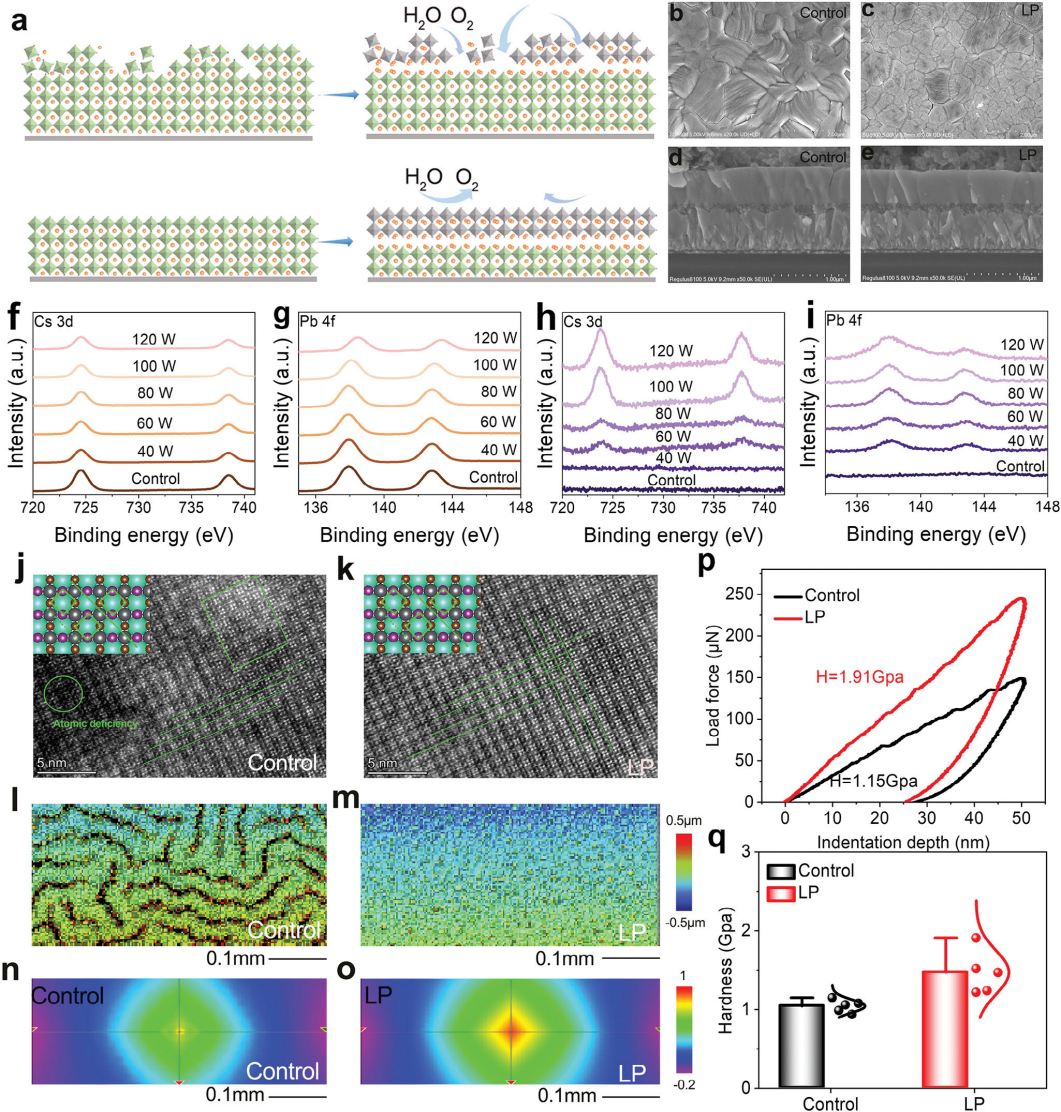
a) Effect of perovskite surface on the formation of 2D/3D heterointerface. b,c) Top‐view and d,e) cross‐sectional SEM images of perovskite films before and after LP treatment. XPS spectra of f) Cs 3d and g) Pb 4f of perovskite films after LP treatment under different powers. XPS spectra of h) Cs 3d and i) Pb 4f for the reclaimed Al_2_O_3_ nanoparticles. HAADF‐STEM images of j) control and k) LP‐treated perovskite films. l,m) 3D optical morphological maps and n,o) autocorrelation function graphs of perovskite films. p) P‐h curves and q) statistic surface hardness of perovskite films.

As illustrated in **Figure** **1**a, the initial surface inhomogeneity highly determines the coverage of in situ capped 2D perovskite layer and the interaction with the underlying 3D perovskite phase. Notwithstanding the great potential of low‐dimension‐induced heterointerface in accelerating charge transfer and stabilizing lattice, the presence of out‐of‐order nanoscale clusters still leads to the coexistence of multiple surface structures, inevitably contributing to the infiltration of water/oxygen molecules along defective boundaries and then structural degradation of photoactive perovskite. In comparison, homogenizing the growth substrate with respect to morphology and composition by polishing the perovskite surface could effectively resolve this challenge. To achieve this purpose, we first immersed the air‐fabricated perovskite film into Al_2_O_3_ nanoparticle‐containing isopropanol solution under ultrasonic bath for surface liquid polishing (LP). By carefully controlling the ultrasonic power, the defective lattice is peeled‐off owing to the mechanical collision. To understand this mechanism, we chose all‐inorganic CsPbI_2_Br film as one prototype. In order to verify the effect of isopropanol on the perovskite film during the LP process, the evolution of the perovskite film under the condition of soaking in isopropanol for over 180 min is shown in Figure S1 (Supporting Information). As can be seen from the picture, there is no color change of the perovskite film, indicating the negligible effect of isopropanol on the perovskite film owing to its low polarity. From the scanning electron microscope (SEM) images shown in Figure 1b,c, the control perovskite surface demonstrates a laminated structure surrounded by deep grain boundary grooves in spite of the full coverage and good crystallization. In contrast, the polished surface shows locally‐ordered grain arrangement and the lamellar structure is removed, along with the significantly reduced roughness (Figure S2a,b, Supporting Information) and narrowed surface potential distribution (Figure S2c—e, Supporting Information). Compared to the solid polishing and other polar solvent induced surface dissolution,^[^ ^28^, ^29^ ^]^ this LP technology has slightly effects on the film thickness (Figure 1d,e), the crystal structure (Figure S3a, Supporting Information) and the light absorption (Figure S3b, Supporting Information), owing to the relatively moderate energy to destroy the whole lattice under the presence of a buffer zone to avoid the significant erosion of perovskite lattice by the solvent with low polarity and solid nanoparticles. Consequently, the carrier generation and transport processes are not affected. The improved crystallization after LP treatment possibly arises from the recrystallization process during soaking treatment induced by isopropanol and the stabilized lattice to withstand the environment during characterization.^[^ ^30^ ^]^ However, for the case treated under the higher ultrasonic power, for example, up to 120 W, the perovskite degrades quickly, and lead iodide‐like flakes and metallic lead clusters start to appear (Figure S4, Supporting Information), which always serve as recombination centers, decreasing the PSC performance.^[^ ^31^, ^32^ ^]^ This is confirmed by X‐ray photoelectron spectroscopy (XPS) spectra. Obviously, as shown in Figure 1f,g and Figure S5 (Supporting Information), after polishing treatment with the increasement of ultrasonic power, there is no change for Cs 3d, Pb 4f, I 3d and Br 3d XPS spectra in perovskite films from 40 to 100 W, an indicator of the only mechanical interaction between Al_2_O_3_ and perovskite lattice without passivation effect because of the weak adhesion ability of Al_2_O_3_ on perovskite surface, while the shift to larger binding energy of Pb 4f in 120 W‐tailored film means the formation of Pb‐related defects, such as PbI_2_ and Pb^0^ (Figure S6, Supporting Information), highly agreeing well with the morphological evolution. Meanwhile, by detecting the blackened Al_2_O_3_ nanoparticles after LP implementation (Figure S7, Supporting Information), the corresponding signals of perovskite are gradually strengthened, as shown in Figures 1h,i, and S8 (Supporting Information), undoubtedly demonstrating the success in polishing the surface for subsequent 2D perovskite coverage.

In order to directly investigate the defect structure, we then performed an atomic resolution high‐angle annular dark field scanning transmission electron microscopy (HAADF‐STEM) test. As shown in Figure 1j,k, the atomic defects and blurry areas that are referred to as amorphous phase regions can be found in the control perovskite surface; In striking contrast, the lattice of the polished surface is tightly packed and uniformly distributed, with no obvious vacancy defects or lattice distortion. Apart from the atomic level, morphological irregularities on the surface resulted from the mismatch between the solvent evaporation rate and Marangoni flow,^[^ ^13^, ^33^, ^34^ ^]^ such as wrinkling and bulging spikes, where the deformation strain and defects are easily propagated, are effectively eliminated (Figure 1l,m), as evidenced by the vanishment of red and black areas in 3D optical morphological maps. Figure 1n,o provides the autocorrelation function images for the upper surface of perovskite films. With LP treatment, the autocorrelation function of the perovskite film decays more slowly along the y‐ and x‐directions, suggesting a smaller variation of the roughness height gradient for the target film. The removal of soft defective layer that plays the role of adhesive tape on the underlying perovskite lattice also benefits the release of lattice strain (Figure S9, Supporting Information), which can be cross‐checked by the emergence of microcracks in the case under high power (120 W, see SEM images in Figure S4, Supporting Information). As a result, the defect density is significantly reduced from 4.89 × 10^15^ to 3.52 × 10^15^ cm^−3^ by recording the space‐charge‐limiting‐current (SCLC) curves (Figure S10, Supporting Information), enabling the enhancement of photoluminescence (PL) intensity (Figure S11a, Supporting Information) and extension of the carrier lifetime (Figure S11b, Supporting Information), which highly determines the charge extraction‐transfer‐recombination behavior and thus the photovoltaic performance of final PSC.^[^ ^35^, ^36^, ^37^ ^]^ Given the soft feature of amorphous surface, we performed nanoindentation measurement of perovskite films before and after LP treatment. From the load‐displacement (P‐h) curves (Figure 1p) that were obtained by indenting the perovskite films with a Berkovich tip at a given indentation depth of 50 nm, a loading force ≈1.5 times higher than that of the pristine film is required (from 150 to 250 µN) for the polished case, which corresponds to an enhancement in hardness. By randomly choosing and measuring five locations on the perovskite surface (Figure S12, Supporting Information), the average hardness of the perovskite surface increases from 1.15 to 1.51 GPa (Figure 1q). Thus, using energetic nanoparticles to scrape the “poison” (defects) off the “bone” (perovskite film) is realized, leaving the advantageous ground for obtaining a closely‐contacted 2D/3D heterointerface.

**Figure 2:**
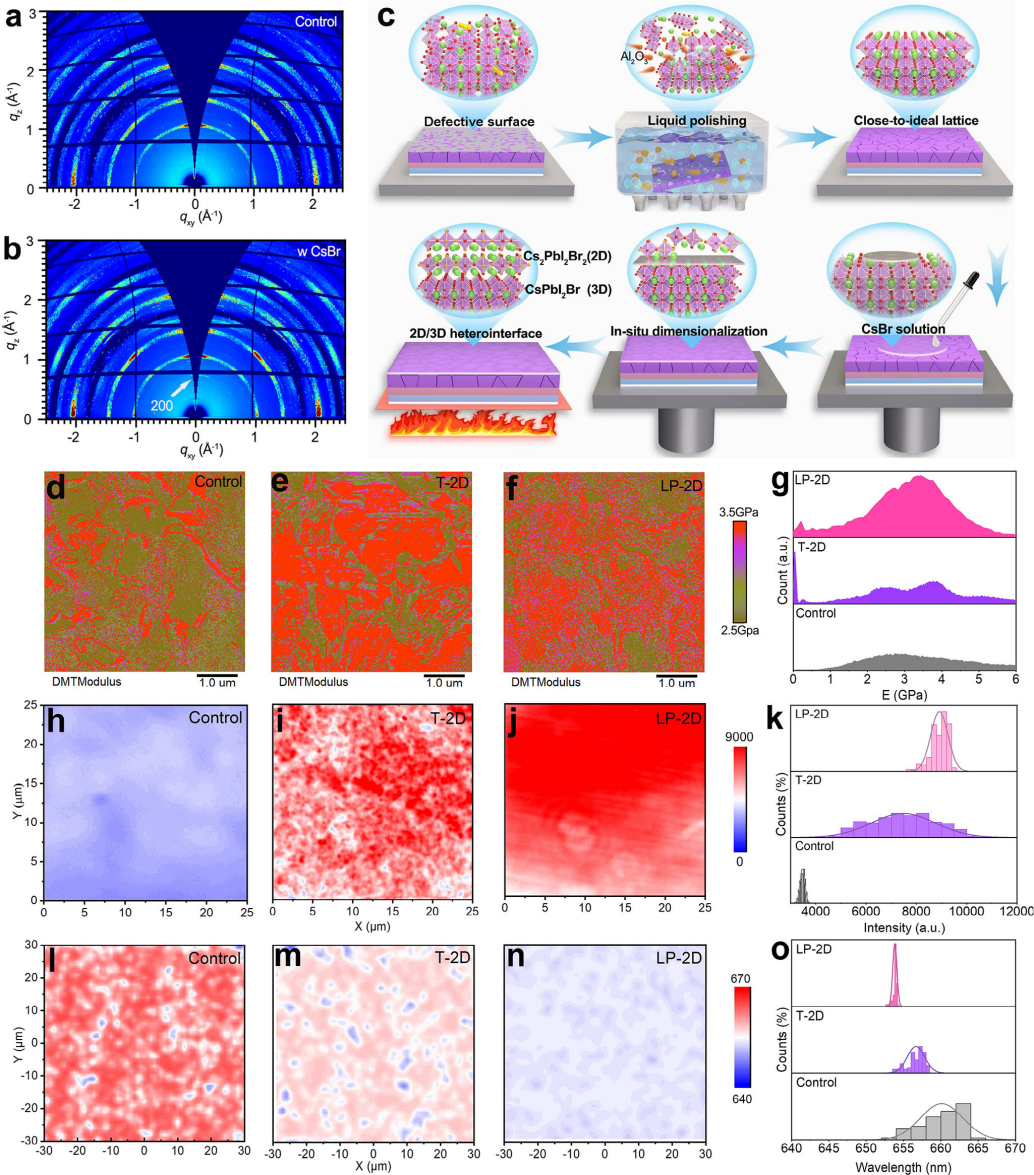
a,b) GIWAX patterns of CsPbI_2_Br perovskite films with and without CsBr treatment. c) Mechanism diagram of the dual‐mechano‐chemical strategy. d–f) DMT moduli maps of various films. g) DMT moduli distribution histograms for perovskite films. h–j) PL mapping images of various perovskite films. k) PL intensity distribution histograms. l–n) The mapping images of PL peak position and o) PL peak position distribution histograms for perovskite films.

Aiming to compensate for the fatigue behavior of organic species, we constructed an all‐inorganic 2D perovskite capping layer to regulate the interfacial energetics and further stabilize the surface by treating the CsPbI_2_Br surface with a CsBr solution, followed by thermal annealing. To understand the feasible formation of structural heterointerface, we first conducted X‐ray diffraction (XRD) measurement of unpolished perovskite films with and without CsBr treatment. But no obvious characteristic peaks corresponding to 2D perovskite phase is observed (Figure S13a, Supporting Information), which is possibly attributed to the ultrathin thickness. By controlling the incidence angle at 0.3° to directly observe the crystal structure of the upper surface of CsPbI_2_Br film (Figure S13b, Supporting Information), two distinct peaks at 9.8° (200) and 17.9° (400) are detected, an indicator of the formation of 2D Cs_2_PbI_2_Br_2_ on the CsPbI_2_Br surface, concomitant to a new absorption peak at wavelength of 565 nm (Figure S13c, Supporting Information). Note that the wide diffraction peak at 10.6–13.8° for the control film mainly comes from the amorphous phase on the surface with substantial atomic‐scale impurities. To further confirm this conclusion, we also performed the grazing‐incidence wide‐angle X‐ray scattering (GIWAXS) patterns, as shown in **Figure** **2**a,b, evidently showing a new emerging peak ≈*q_z_* = 0.71 Å^−1^ after CsBr treatment (that is absent in control case), which undoubtedly corresponds to the (002) and (004) reflections of 2D Cs_2_PbI_2_Br_2_ perovskite.^[^ ^38^, ^39^ ^]^ Increasing CsBr concentration results in an increase in the relative intensity of two reflections (Figure S14, Supporting Information), showing the successful implementation of a thin 2D inorganic perovskite capping layer.

Based on the abovementioned conclusions, we combined the LP technique and dimensionality control to idealize the surface, denoted as a dual‐mechano‐chemical strategy illustrated in Figure 2c, and then explored the evolution of surface dimensional homogenization. For comparison, we mainly focused on the control film, traditional 2D perovskite‐capped film without LP treatment (T‐2D), and the case after LP treatment (LP‐2D), respectively. By further recording the SCLC curves of perovskite films coated with 2D phase, the defect density is gradually reduced, following the order of the control (4.89 × 10^15^ cm^−3^) > T‐2D (3.99 × 10^15^ cm^−3^) > LP‐2D (3.27 × 10^15^ cm^−3^) (Figure S15, Supporting Information). This result directly confirms the advantage of LP treatment on following fabrication of a robust 2D/3D heterointerface for healing defects. Depositing the surface morphology after dimensionalization is nearly unchanged (Figure S16, Supporting Information), this approach highly determines the mechanical and electrooptical homogenization of 2D/3D heterointerface. We first performed the peak force quantitative nanomechanical atomic force microscopy (PFQNM‐AFM) measurement to characterize the mechanical response of perovskite films by extracting the average Derjaguin–Müller–Toporov (DMT) moduli and distribution. From the modulus maps shown in Figure 2d, the multi‐colored surface textures for the control film demonstrate a heterogeneous modulus distribution owing to the out‐of‐order distribution of soft defects located at the surface.^[^ ^30^, ^40^ ^]^ With in situ surface dimensional reconstruction, an increase in Young's modulus from 2.760 GPa for the pristine film to 3.545 and 3.189 GPa for the 2D Cs_2_PbI_2_Br_2_ tailored samples with and without surface polishing is observed (Figure 2e,f), confirming the higher mechanical hardness because of the removal of amorphous phase. In spite of this, it should be noted that, as for the traditional 2D‐capped film, there is still a substantial variation in modulus across the sample, which stems from the inhomogeneous nano‐impurities as templates for 2D perovskite growth. Fortunately, this scenario is significantly weakened by first homogenizing the defective surface prior to engineering dimensionality, as evidenced from the narrowed modulus distribution (Figure 2g). Such a homogenous lattice could lead to a more stable heterointerface for withstanding external stimuli, along with reduced defects.

Apart from the mechanical properties of perovskite films, we also evaluated the 2D perovskite heterogeneity with respect to the spatial and spectral variations in the PL peak intensity of the perovskite surface. As shown in Figure 2h–k, a hyperspectral PL mapping was performed for the perovskite surface treated by various methods. Obviously, both perovskite films capped with 2D layer exhibit a PL intensity double higher than that of the control film, highly indicative of suppressed nonradiative recombination owing to the reduced defect.^[^ ^41^, ^42^ ^]^ More importantly, in agreement with the substantial improvement in the surface mechanical uniformity, this dual‐mechano‐chemical strategy also homogenizes the PL intensity with the narrowest distribution. In general, the mechanism behind PL heterogeneity can be arisen from defects and phase variations.^[^ ^43^ ^]^ Therefore, to investigate phase heterogeneity, we further recorded the PL peak position maps (Figure 2l‐o). In contrast to the broader distribution observed in control case and even in only T‐2D‐capped film, the LP‐2D‐capped film exhibits a narrower distribution in PL peak positions predominantly centered at 654 nm, accompanied by a blue‐shifted emission wavelength, which closely aligns with the passivated defect by 2D Cs_2_PbI_2_Br_2_ as well as the removal of more I‐rich phase on the surface because of the weaker Pb‐I bond than that of Pb‐Br bond.^[^ ^44^ ^]^ Based on the above characterizations, we can conclude that a more homogeneous and lower‐defect perovskite interface is obtained, which benefits the maximization of 2D capping layer on solidifying and protecting the lattice.

**Figure 3:**
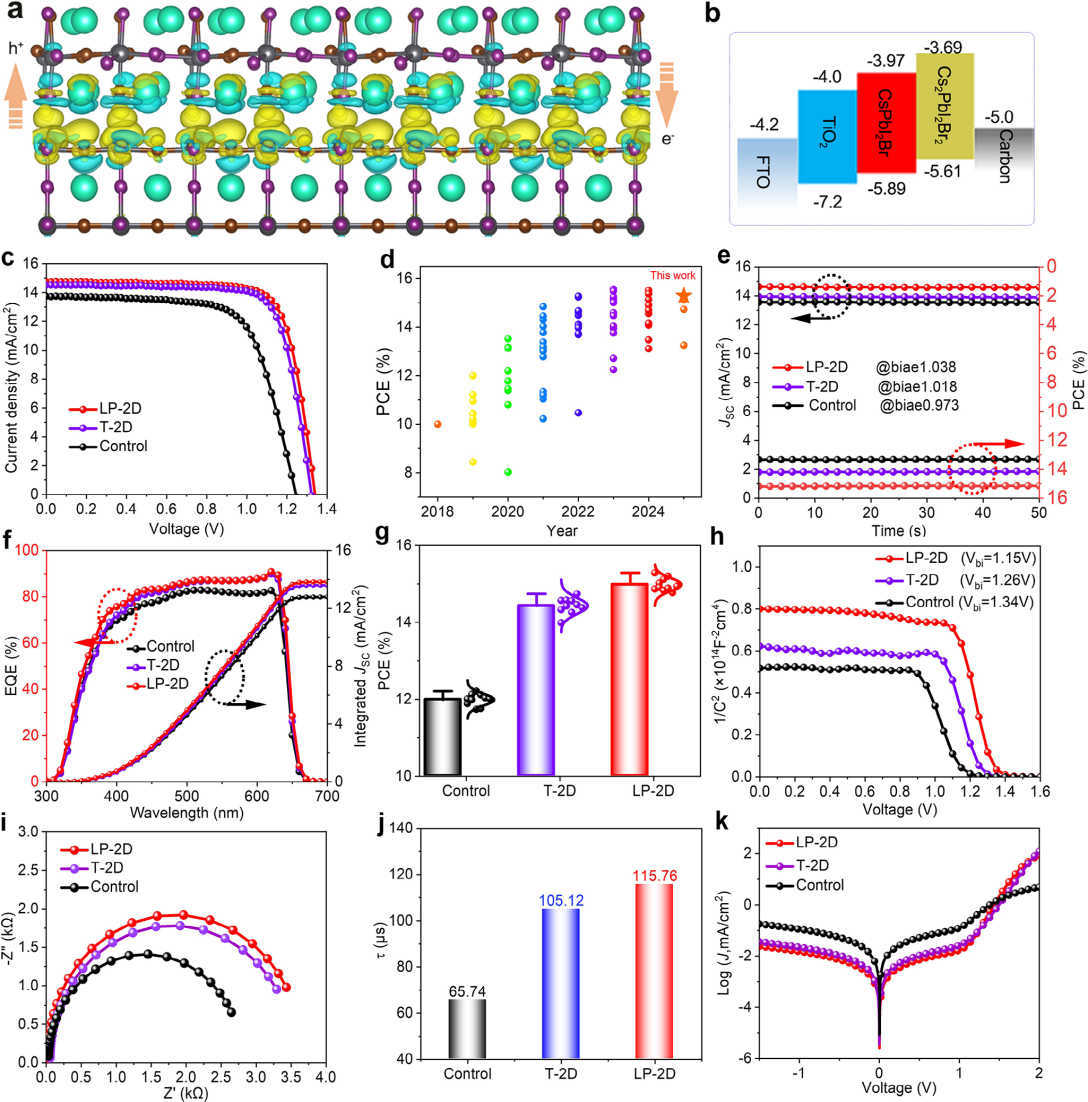
a) Calculated charge density difference for Cs_2_PbI_2_Br_2_/CsPbI_2_Br heterojunction. b) Energy level diagram of PSCs based on perovskite with and without CsBr treatment. c) *J–V* curves of CsPbI_2_Br PSCs. d) Statistical PCEs of reported carbon‐based CsPbI_2_Br PSCs fabricated in air condition. e) Steady power outputs under bias at maximum power points, f) EQE spectra, g) statistical PCEs for various PSCs. h) Mott‐Schottky curves, i) electrochemical impedance spectra, j) electron lifetime, and k) dark *J–V* curves of control, T‐2D and LP‐2D perovskite tailored PSCs.

In a PSC, charge transfer dynamics at 2D/3D heterointerface also correlate to the photovoltaic performance. To understand the positive effect of this 2D Cs_2_PbI_2_Br_2_ layer on photogenerated carrier extraction, we preferentially calculated the charge density profiles based on density functional theory simulation by building the periodic slabs of CsPbI_2_Br and Cs_2_PbI_2_Br_2_ perovskite. After relaxing the atom position, we observe that the positive charges (yellow region) are mainly located at the interface near 3D slabs, while the negative charges (blue region) are distributed near 2D slabs (**Figure** **3**a). This agrees well with the reported results previously.^[^ ^45^ ^]^ In other words, the 2D/3D heterointerface promotes the photogenerated carrier (hole) extraction from the underlying CsPbI_2_Br to the upper Cs_2_PbI_2_Br_2_ layer, which can be cross‐checked by the energy level alignment obtained from ultraviolet photoelectron spectroscopy (UPS) spectra (Figures 3b and S17, Supporting Information). Following this line of thought, surface dimensional homogeneity is expected to strengthen the charge transfer and extraction behavior, as evidenced by a significant decrease in PL intensity and carrier lifetime by testing the glass/perovskite/carbon sample from the glass side (Figure S18, Supporting Information).^[^ ^46^, ^47^, ^48^ ^]^

We then fabricated solar cells in complete air condition, with a typical architecture of FTO/TiO_2_/perovskite/carbon to explore device performance and longevity. Figure 3c shows the champion photocurrent density‐voltage (*J‐V*) curves of various devices after CrBr concentration optimization (Figure S19, Supporting Information) and the corresponding photovoltaic parameters are summarized in Tables S1,S2 (Supporting Information). Owing to the more susceptible surface for the air‐processed CsPbI_2_Br film, the control device only delivers a short‐circuit current density (*J*_SC_) of 13.77 mA cm^−2^, an open‐circuit voltage (*V*_OC_) of 1.245 V, a fill factor (FF) of 68.95%, and a PCE of 11.82%, while an enhanced PCE of 14.80% is achieved by in situ constructing traditional 2D/3D heterointerface. It should be noted that only isopropanol treatment also slightly improves the PCE to 12.67% (Figure S20, Supporting Information), which can be attributed to the removal of surface defects or recrystallization inspired by our previous study, in line with the increased XRD peak (Figure S3, Supporting Information).^[^ ^30^ ^]^ With the improved uniformity and strengthened contact, the target device shows a champion PCE of 15.29%, with a *J*_SC_ of 14.81 mA cm^−2^, a *V*_OC_ of 1.34 V, and an FF of 77.00%, which is one of the highest values among the congeneric CsPbI_2_Br devices reported so far (Figure 3d).^[^ ^49^, ^50^, ^51^, ^52^, ^53^ ^]^ Combining the stable PCE of 15.15% and integrated photocurrent density of 13.85 mA cm^−2^ for target device that highly agrees well with the values obtained from *J‐V* curves by recording stabilized power outputs (SPO, Figure 3e) and external quantum efficiency spectra (EQE, Figure 3f), we can confirm the validity of the improved performance by this dual‐mechano‐chemical strategy. These results are also in line with the PCE statistics shown in Figure 3g, a consequence of the homogeneous dimensionality on the surface that effectively reduces defect and accelerates charge extraction.

To get deep insights into the recombination losses within a device, we systematically studied the charge carrier dynamics, including the charge transport and recombination behaviors, using popularly used electrochemical techniques. Based on the capacitance‐voltage (C‐V) measurement (Figure 3h), a higher built‐in electric field (*V*_bi_) of 1.34 V for LP‐2D tailored device than that of 1.15 and 1.26 V for control and T‐2D‐capped devices means the larger driven force for photogenerated carrier separation rather than direct recombination, which is compatible with the higher *V*_OC_ and in accordance with the increased recombination resistance (Figure 3i). Meanwhile, we recorded the dependence of *J*_SC_/*V*_OC_ on light intensity to reveal the recombination mechanism. After calculation, the ideality factors are closer to unit value (Figure S21, Supporting Information), an indicator of the simultaneous suppression of bimolecular recombination (short‐circuit state) and defect‐assisted Shockley‐Read‐Hall recombination (open‐circuit state). The reason behind these results mainly attributes to the reduced surface defects and matchable band alignment, which can be also cross‐checked by the prolonged decay time of photovoltage in LP‐2D perovskite film from initial 65.74 to 115.76 µs (Figure 3j and Figure S22, Supporting Information), leading to the reduced leakage current in target device (Figure 3k).

**Figure 4:**
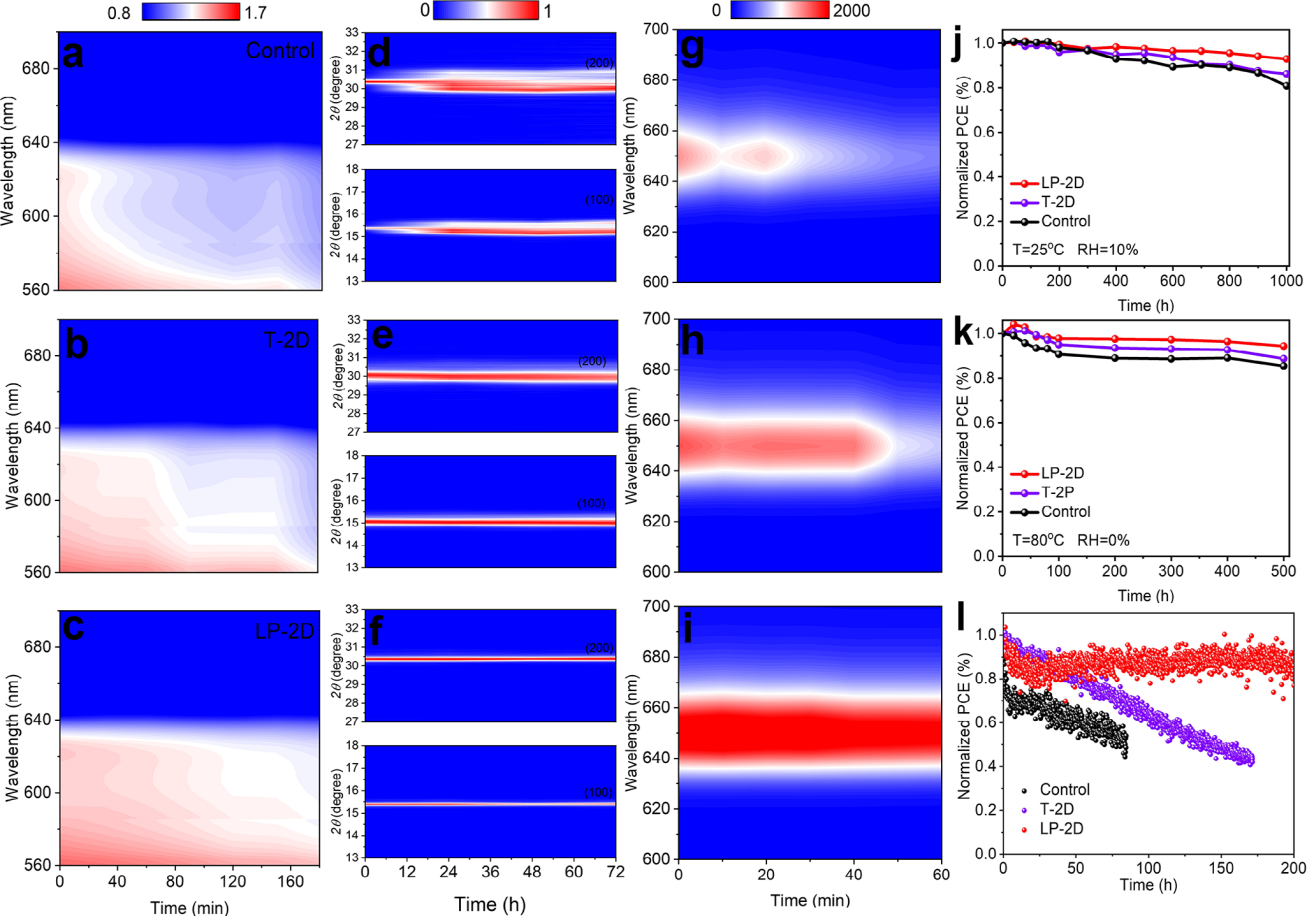
a–c) Evolution of the absorbance change of perovskite films in air condition over 180 min. d–f) Evolution of (100) and (200) planes of perovskite films in air condition over 72 h. g–i) Evolution of PL spectra of perovskite films in air condition over 60 min. Normalized PCE of unencapsulated PSCs under j) air condition with temperature of 25 °C and RH of 10%, k) high temperature of 80 °C and l) persistent light irradiation.

Finally, we evaluated the impacts of the dimensionality homogenization on device durability under various conditions. Prior to monitoring the device efficiency, we first examined the time‐dependent properties of perovskite films. Although 2D Cs_2_PbI_2_Br_2_ perovskite has an imperceptible effect on water contact angle (Figure S23, Supporting Information), the film with LP‐2D capping layer fades obviously slower than the control and even T‐2D‐capped films after storage in air condition without any protection for over 180 min (Figure S24, Supporting Information), showing excellent resistance against moisture. It is undoubtable that the homogeneous coverage of 2D perovskite should be responsible for this phenomenon. To reveal the structural evolution, we collected the absorption spectra and XRD patterns of perovskite films at various time intervals. As shown in **Figures** **4**a‐c and S25 (Supporting Information), the characteristic absorption peak centered at 625 nm for the control film is gradually quenched within 180 min due to the moisture‐induced decomposition of the perovskite phase. On the contrary, the incorporation of 2D capping layer, especially with additional liquid polishing process, effectively weakens this adverse transformation, an indicator of the solidified lattice to withstand external stimulus. This result is further authenticated by the evolution of XRD patterns in Figure 4d‐f and Figure S26 (Supporting Information). After homogenizing surface dimensionality, obviously, the target film shows an almost constant width at half‐height for the diffraction peaks of (100) and (200) planes (pure CsPbI_2_Br phase) after aging ≈72 h in air, while the control film exhibits a fast increase and even gradually splits into two peaks. Agreeing well with the above conclusions, the PL intensity that highly depends on perovskite phase reasonably suffers from rapid quenching for control sample, and a moderate loss for T‐2D‐capped film owing to the insufficient protection capacity, whereas no noticeable change is observed for LP‐2D‐capped perovskite (Figures 4g‐i and S27, Supporting Information), again demonstrating the great superiority of homogenizing surface dimensionality on stabilizing perovskite lattice.

As is well known, the film quality highly determines the final PSC longevity. When stored in ambient air with relative humidity (RH) of 10% and room temperature (T) of 25 °C, as shown in Figures 4j and S28 (Supporting Information), the unencapsulated device with homogenous surface demonstrates the largest PCE conservation rate (92.9%) compared to control device (80.8%) and T‐2D‐tailored device (86.2%) after 1000 h of storage. Meanwhile, thermal accelerated aging measurements were also conducted on three unencapsulated devices under low‐vacuum conditions at 80 °C, which has been shown to be more severe than an inert environment for perovskite degradation.^[^ ^54^ ^]^ After storage for over 500 h, the target PSC still maintains 94.4% of initial efficiency, while the PCE of the control device drops to 89% and that of T‐2D case decreases to 85.51% (Figure 4k and Figure S29, Supporting Information). Similarly, by subjecting the unencapsulated devices to the maximum power point tracking (MPPT) condition under one‐sun‐equivalent white LED light illumination with temperature of 25 °C for 200 h (Figure 4l), we obtain the same tendency, maintaining 83.3% of its initial PCE for target device, but experiencing rapid decay to 49.6% within only 84 h and 42% within 170 h for control and T‐2D devices, respectively. All these observations corroborate the ability of homogenizing 2D/3D heterointerfaces to strengthen the tolerance of PSCs to multiple environments.

## 3 Conclusion

In summary, we demonstrate a dual‐mechano‐chemical strategy to precisely homogenize 2D/3D heterointerfaces by combining energetic polishing and dimensionality engineering for targeting the significant improvement on stability and efficiency of PSCs. Compared to the traditional heterointerface, the first removal of defective nano‐impurities and micro‐wrinkles that flattens the groundwork for upper surface manipulation can weaken the dimensional complexity and heterogeneity from mixed phases, leading to a strengthened heterointerface with matchable energy alignment and defect‐free feature, thus effectively stabilizing the lattice and minimizing the recombination loss. As a result, with the implementation of a close‐knit Cs_2_PbI_2_Br_2_/CsPbI_2_Br surface, we obtain an all‐air‐processed carbon‐based CsPbI_2_Br PSC with enhanced efficiency of 15.29%, more importantly, accompanied by well‐improved durability under harsh conditions, such as high humidity, high temperature, and persistent light irradiation. This work not only highlights the great importance of surface dimension regulation on PSC performance and stability, but also provides a strategic design for promoting the availability of air‐processed perovskite devices.

## Conflict of Interest

The authors declare no conflict of interest.

## Supporting information



Supporting Information

## Data Availability

Research data are not shared.
