# Supplementary material for: Homogeneously‐Dimensionalizing Perovskite Surface by Dual‐Mechano‐Chemical Regulation for Efficient Solar Cells
Source: Adv Sci (Weinh). 2025 Jul 13;12(38):e09089. doi: 10.1002/advs.202509089 (PMC12520521; doi:10.1002/advs.202509089)
Supplement: Supplementary file 1 — Supporting Information [file ADVS-12-e09089-s001.doc]

Supporting Information

**Homogeneously-Dimensionalizing Perovskite Surface by Dual-Mechano-Chemical Regulation for Efficient Solar Cells**

*Shengwei Geng, Jialong Duan,* Chenlong Zhang, Jinyue Zhang, Yueyang Bi, Yueji Liu, Xixi Zhu,* Xinyu Zhang, Qiyao Guo, Jie Dou, Benlin He, Yuanyuan Zhao, and Qunwei Tang**

1. Geng, Prof. J. Duan, C. Zhang, J. Zhang, Y. Bi, Y. Liu, Prof. X. Zhu, Prof. X. Zhang, Prof. Q. Guo, Prof. J. Dou, Prof. Q. Tang
   College of Chemical and Biological Engineering

Shandong University of Science and Technology

Qingdao 266590, PR China

E-mail: duanjialong@sdust.edu.cn; zhuxixi@sdust.edu.cn; tangqunwei@sdust.edu.cn.

S. Geng
Wuhan National Laboratory for Optoelectronics and School of Physics

Huazhong University of Science and Technology

Wuhan 430074, PR China

Prof. B. He
School of Materials Science and Engineering

Ocean University of China

Qingdao 266100, PR China

Prof. Y. Zhao
College of Energy Storage Technology

Shandong University of Science and Technology

Qingdao 266590, PR China

Experimental Details

**Materials and Reagents**

Cesium iodide (CsI, 99.99%), lead bromide (PbBr_2_, 99.99%), cesium bromide (CsBr, 99.99%), anhydrous dimethyl sulfoxide (DMSO, 99.7%), aluminum oxide (the size is 30 nm), diethanol amine (99%), titanium (IV) isopropoxide (99.9%), isopropanol (IPA, 99.5%) and methyl alcohol (MeOH, 99.5%) was purchased from Aladdin. Lead iodide (PbI_2_, 99.99%) were obtained from Xi’an Yuri Solar Co., Ltd. Zinc powder and hydrochloric acid were obtained from AR Guangzhou Chemical Reagent Factory. Commercial carbon paste was obtained from Shanghai MaterWin New Materials (MTW-CE-C-003, ≈10 Ω sq^−1^). Fluorine-doped tin oxide coated (FTO) substrates with a sheet resistance of 14 Ω sq^−1^ were purchased from Pilkington. Unless mentioned otherwise, all the materials and reagents were commercially purchased and used as received free of further purification.

**Fabrication of Solar Cells**

FTO was first etched with the desired pattern via zinc powder and hydrochloric acid and then consecutively washed with deionized water, acetone, isopropanol, and ethanol in an ultrasonic bath for 30 min, respectively, and finally treated with UV-ozone for 180 s. A compact titanium dioxide layer (c-TiO_2_) was prepared by spin-coating a solution (0.5 M diethanol amine and 0.5 M titanium isopropoxide ethanol solution, respectively) at 7000 rpm for 30 s, and then annealed at 500 ^o^C for 2 h in air. Prior to transferring the cooled substrates into N_2_ glove box, the FTO/c-TiO_2_ substrates were treated with UV-ozone for another 180 s. CsPbI_2_Br perovskite layer was deposited via a two-step thermal treatment process. Briefly, 80 μL of 1.2 M CsPbI_2_Br precursor solution containing 312 mg of CsI, 220 mg of PbBr_2_, 277 mg of PbI_2_, dissolved in 1 mL DMSO was spin-coated onto FTO/c-TiO_2_ substrate at 1000 rpm for 10 s and then 3000 rpm for 30 s. A hot flow of 100 ^o^C was blown onto the substrate until the color of the film turned brown. The distance between the muzzle of hot-air gun (858D, DongGuan BuFan Electronics Co., LTD) and the substrate was ≈ 4 cm. The whole blowing process lasted ≈ 20 s. The prepared perovskite films were mechanically polished in an isopropyl alcohol polishing solution containing aluminum oxide under various powers. The perovskite film after mechanical polishing were washed three times with isopropyl alcohol, and then 2D/3D heterojunction surfaces were in-situ constructed on the upper surface of the perovskite films with CsBr solution (IPA : MeOH = 2 : 1). Afterward, the substrate was placed on a hotplate at 160 ^o^C for 1 min and then at 270 ^o^C for 10 min and cooled down to room temperature naturally. Finally, the commercial carbon paste was bladed onto the perovskite film with an active area of 0.09 cm^2^ and then dried at 200 ^o^C for 2 min.

**Characterizations**

The morphologies were conducted by scanning electron microscopy (SEM, Regulus8100, JAPAN). The ultrathin carbon film-coated copper grid was attached to the glass substrate by polyimide tape, and the substrate was cleaned with ethanol solution and ozone treatment for 8 min before preparing the perovskite thin film for HAADF-STEM characterization. The perovskite thin films were spin-coated from the precursor solution at 1000 rpm for 10 s and then 3000 rpm for 30 s followed by annealing process. Last, the copper grid was carefully clipped from the glass substrate and then loaded into TEM chamber for the characterization. X-ray photoelectron spectroscopy (XPS) spectra were measured via a RBD upgraded PHI-5000C ESCA system (Perkin Elmer) equipped with Mg Kα as the X-ray source (hv = 1253.6 eV). The crystallinity of perovskite films was determined by the X-ray diffraction (XRD, Bruker D8 ADVANCE) with Cu Kα (λ = 1.5406 Å) radiation at 40 kV and 40 mA. The optical absorption spectra of perovskite films were characterized by UV-vis spectrophotometer (UV-8000A) under the wavelength range of 300-800 nm. Steady-state photoluminescence (PL) spectra and time-resolved photoluminescence (TRPL) spectra were obtained on a FluoroMax-4 spectrofluorometer and a Horiba spectrometer under excitation wavelength of 410 and 470 nm, respectively. External quantum efficiency (EQE) spectra of devices were obtained by a IPCE kit developed by Enli Technology Co., Ltd. with a standard Si crystalline solar cell as a reference. Current density-voltage (*J-V*) curves of solar cells were measured on an electrochemical workstation (CHI660E) under standard solar irradiation (Enli, Oriel Class A, AM 1.5G, 100 mW cm^−2^, calibrated by a standard silicon solar cell) equipped with 500 W Xenon lamp. Steady-state output of photocurrent and PCE were measured via an electrochemical workstation (CHI660E) under a certain bias. The electrochemical impedance spectroscopy (EIS) measurements and the Mott-Schottky plots, dark *J*-*V* curves were conducted on the CHI660E electrochemical workstation. The light stability was performed under constant irradiation by a LED lamp in the air. All the moisture and thermal durability were measured on the devices without encapsulation.

**Statistical Analysis**

All statistical analyses were performed with Origin 2021. The data obtained from SEM, UV-vis, PL, TRPL, XPS, XRD, *J-V*, EIS and EQE spectra were the original data without normalization. The trap state density (*N*_t_) was calculated according to the space-charge-limited current (SCLC) method with the following equation: *N*_t_ = 2ɛ_0_ɛ_r_*V*_TFL_/*qL*^2^, where *V*_TFL_ is the onset voltage of the trap-filled limit, *q* is the elementary charge of an electron, *L* is the thickness of the perovskite film, and ɛ_0_ is the vacuum permittivity and ɛ_r_ the relative dielectric constant. Bi-exponential decay function was employed to TRPL decays to explore the carrier transport dynamics. Ten PSCs were used to generate the device performance statistics. The summarized champion PCEs for PSCs were obtained from reported works.

**DFT Method**

Density function theory calculation was performed by using the CP2K package. Perdew Burke-Ernzerhof (PBE) functional with Grimme D3 correction was used to describe the system. Kohn-Sham DFT has been used as the electronic structure method in the framework of the Gaussian and plane waves method. The Goedecker-Teter-Hutter (GTH) pseudopotentials, DZVP-MOLOPT-GTH basis sets were utilized to describe the molecules.

Results and Discussion


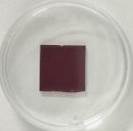

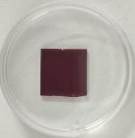

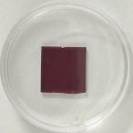

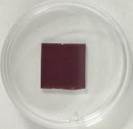

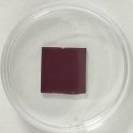

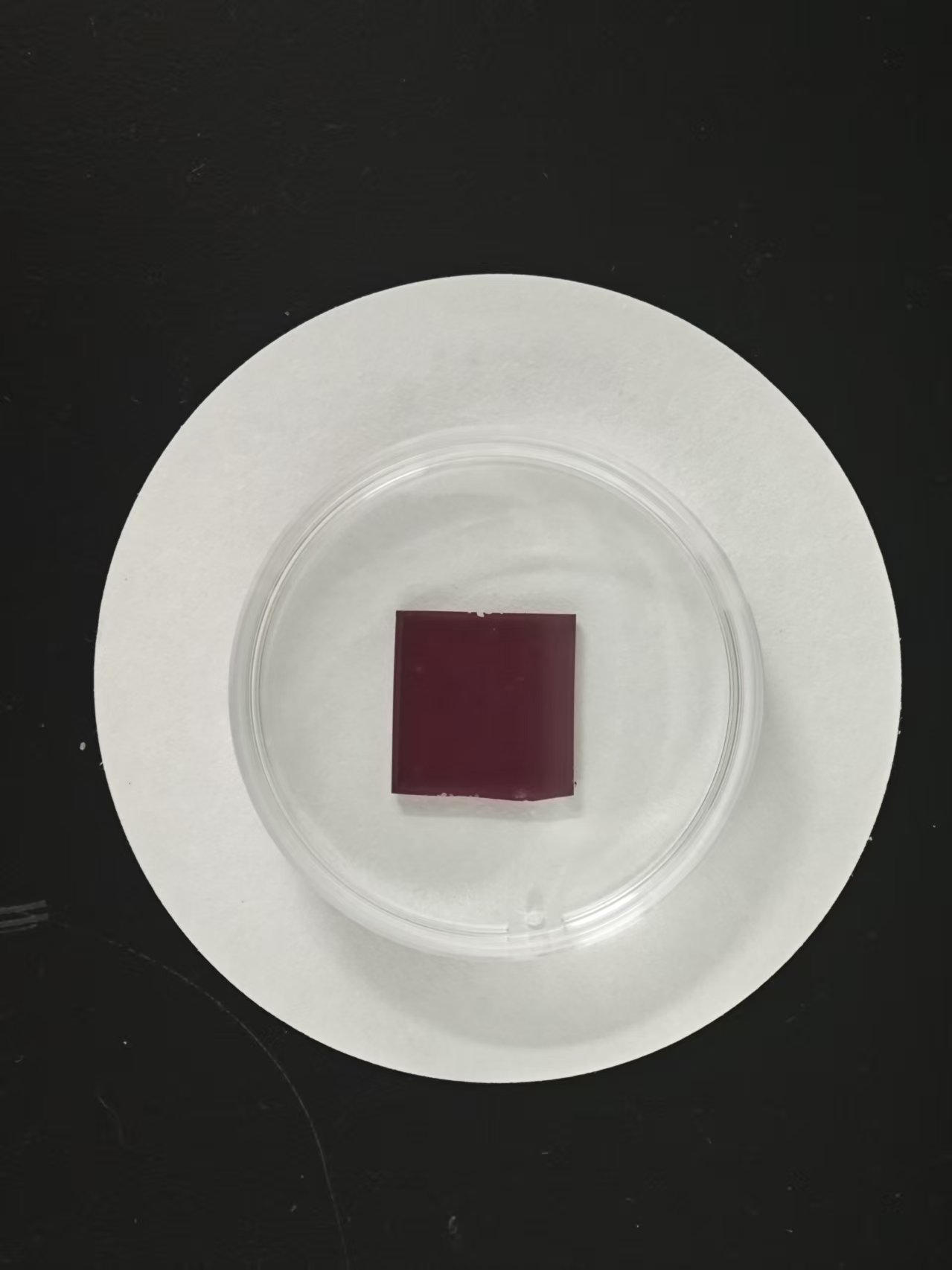

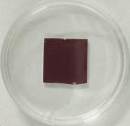

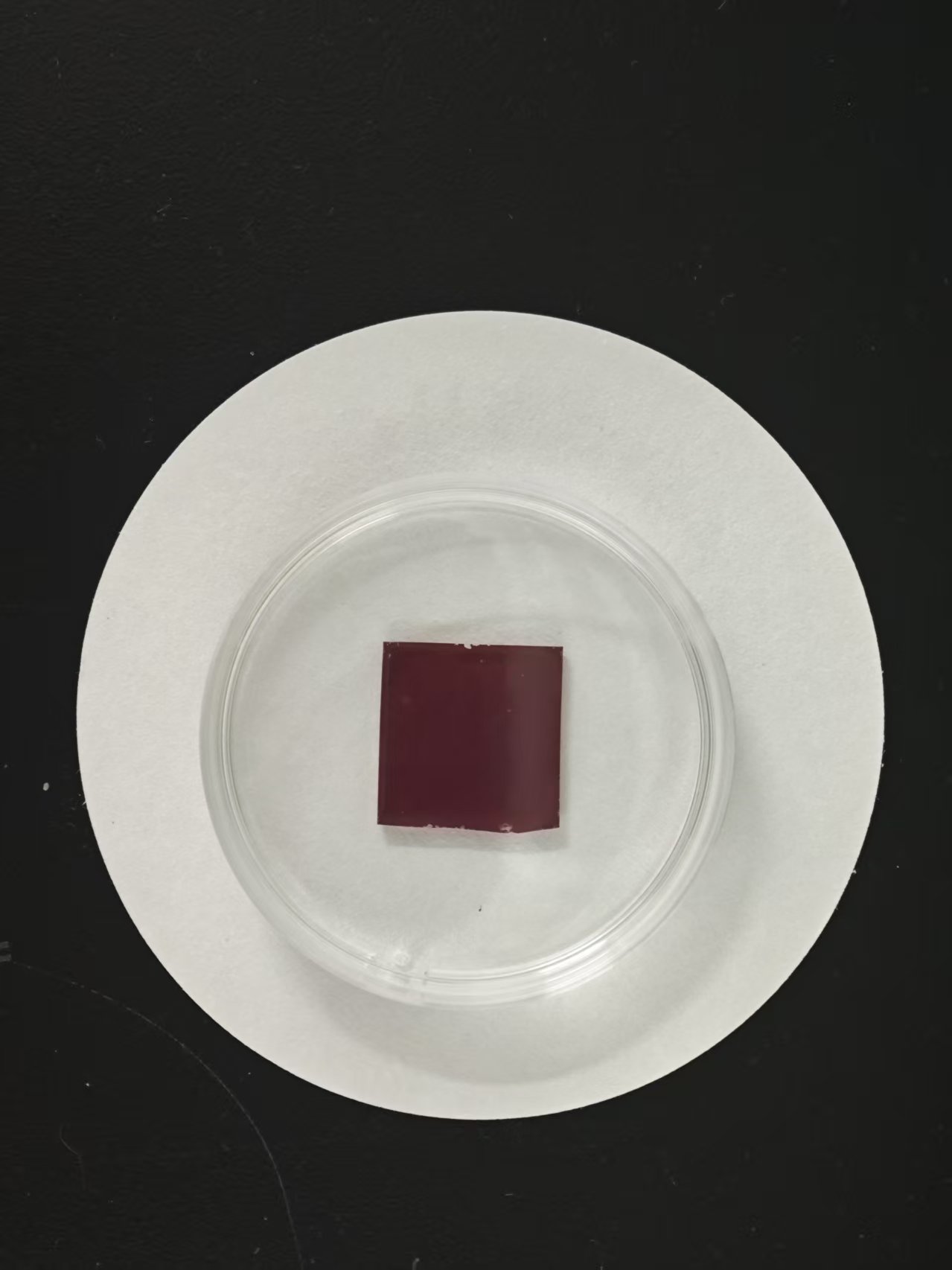

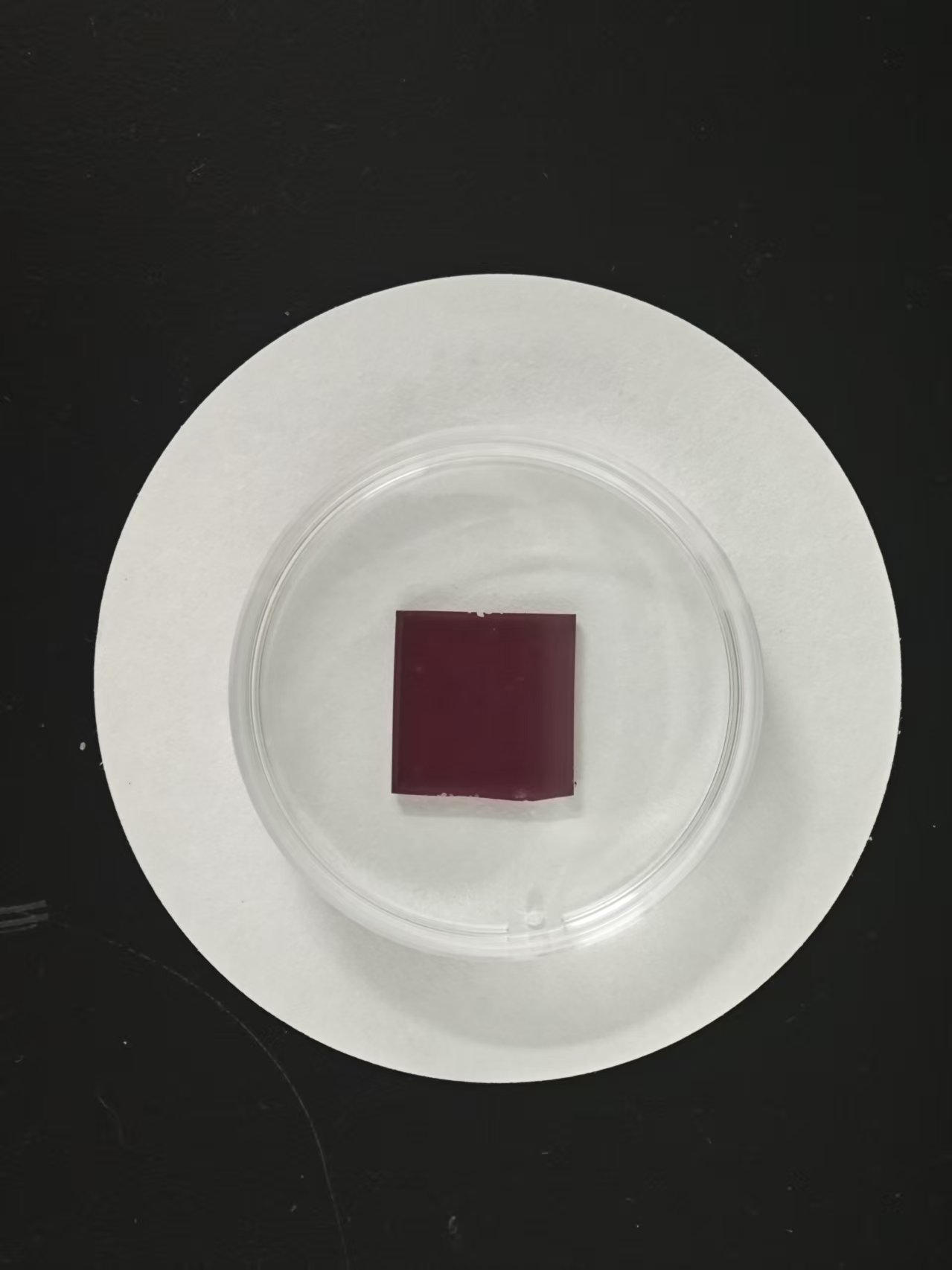

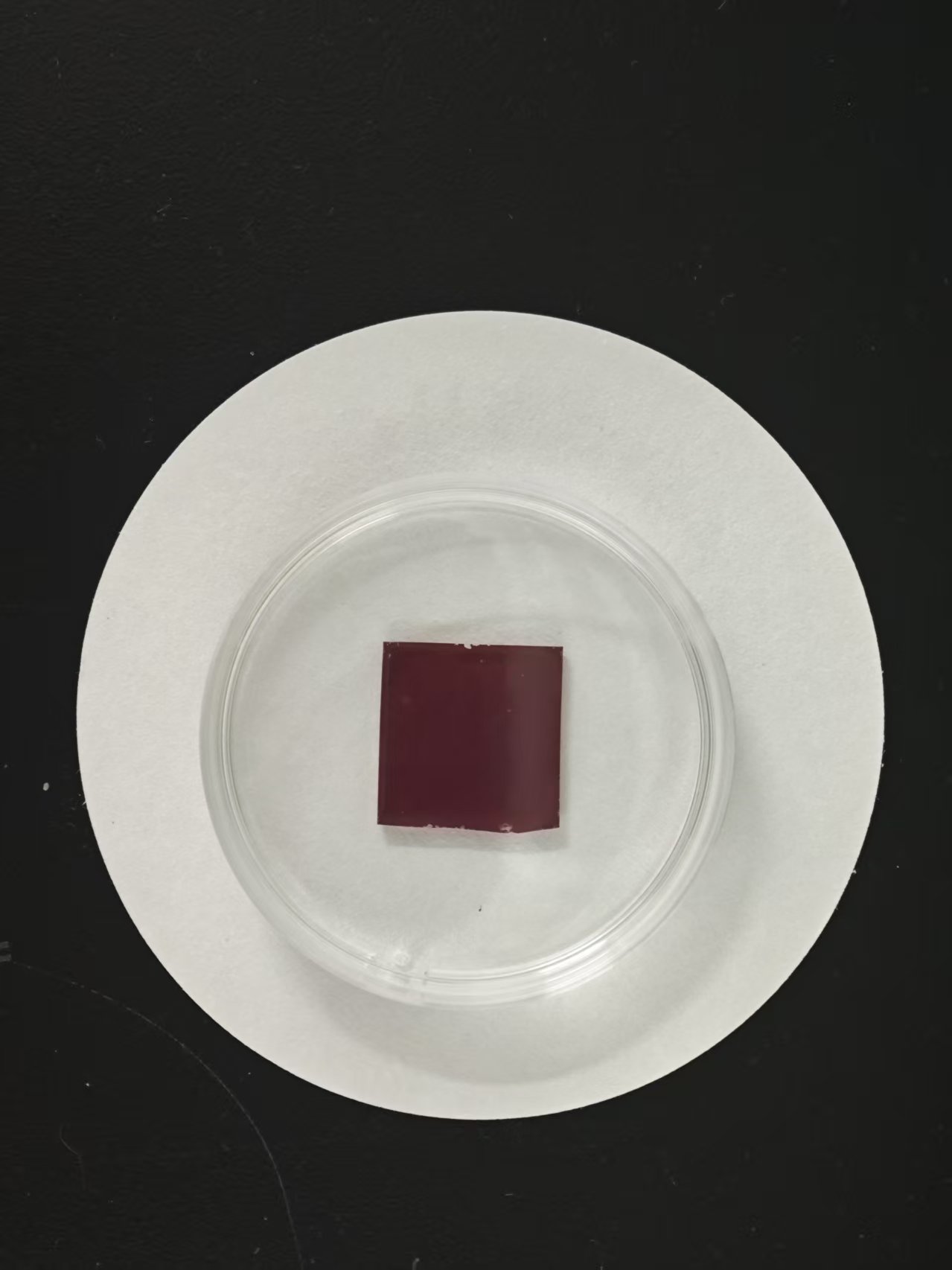

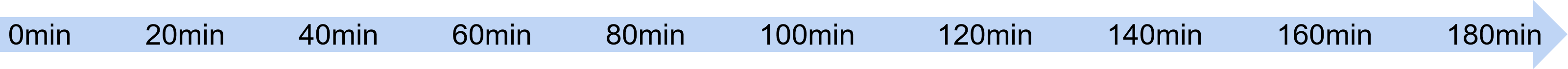


**Figure S1.** Evolution of perovskite film under the condition of soaking in isopropanol over 180 min.


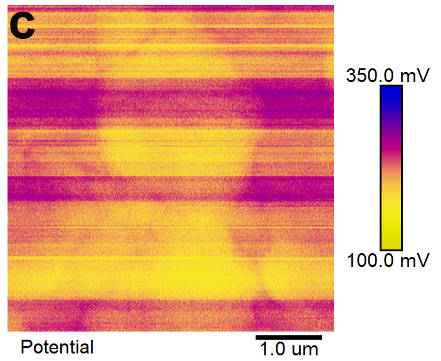

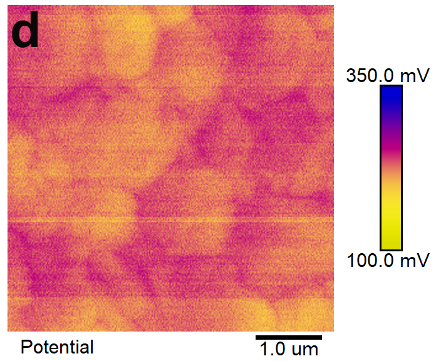

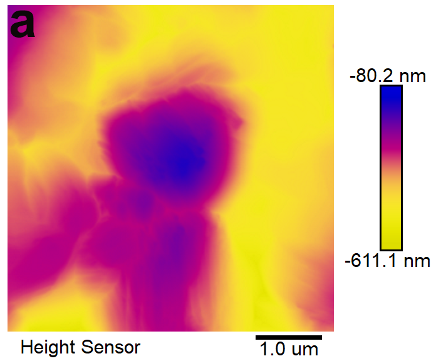

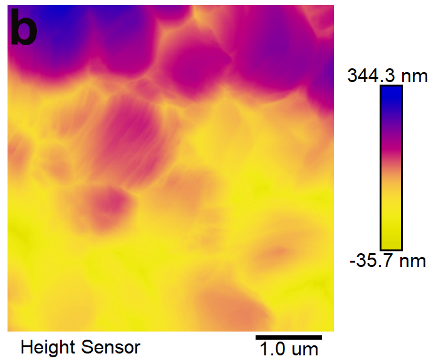


**Figure S2.** AFM height images and KPFM images of (a,c) control and (b,d) LP-treated CsPbI_2_Br films. (e) The surface potential distributions.

**Figure S3.** (a) XRD patterns of control and LP treated CsPbI_2_Br films. (b) Evolution of the absorbance change of perovskite film with different ultrasonic power treatment.


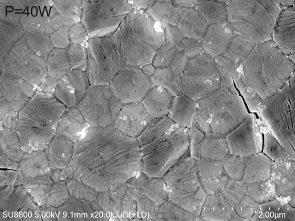

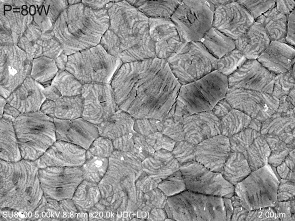

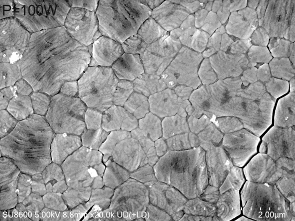

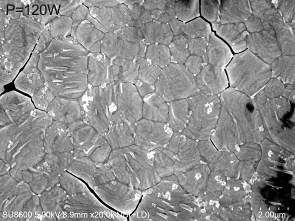


**Figure S4.** SEM images of control and target perovskite films treated under different powers.

**Figure S5.** The XPS peak of I 3d and Br 3d in perovskite films.

**Figure S6**. Pb 4f XPS spectrum of perovskite film after 120 W treatment.


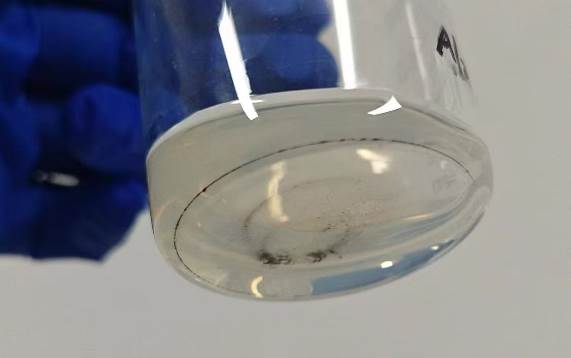


**Figure S7.** The blackened Al_2_O_3_ nanoparticles after LP process.

**Figure S8.** XPS spectra of I3d and Br 3d in Al_2_O_3_ nanoparticles after polishing treatment.

**Figure S9.** GIXRD spectra of perovskite films on TiO_2_ substrates.

**Figure S10.** SCLC curves of electron-only devices based on control and target perovskite films after LP treatment.

**Figure S11.** (a) PL spectra and (b) TRPL decay curves of perovskite films.

**Figure S12.** Nanoindentation curves of perovskite films.

**Figure S13.** (a) XRD patterns, (b) GIXRD curves and (c) UV-vis spectra of unpolished perovskite films on TiO_2_ substrates with and without CsBr treatment.


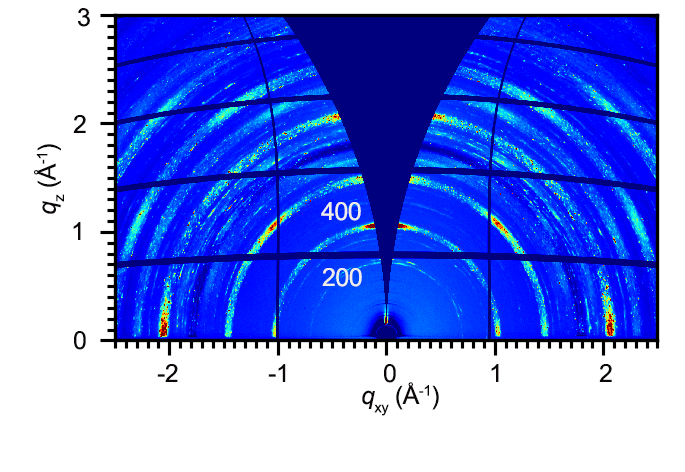


**Figure S14.** GIWAXS characterization of the perovskite film with high concentration of CsBr.

**Figure S15.** SCLC curves of electron-only devices based on control and target perovskite films capped with 2D perovskite layer.


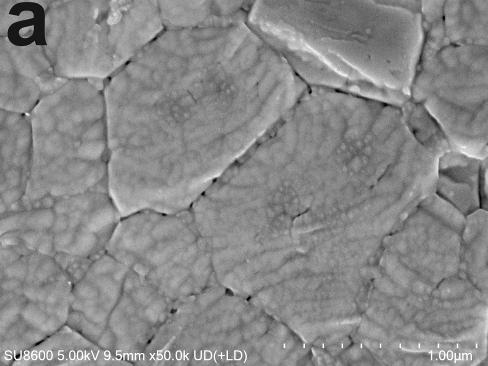

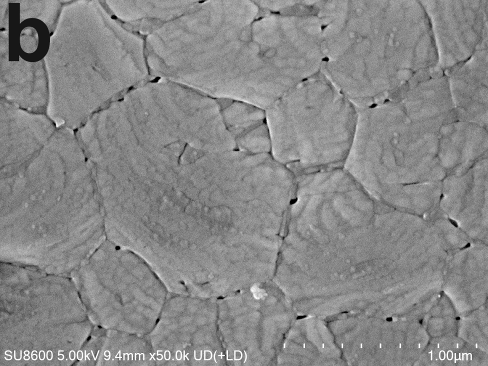


**Figure S16.** SEM images of polished perovskite films without and with CsBr treatment.

**Figure S17.** UPS spectra of perovskite films without and with CsBr treatment.

**Figure S18.** (a) PL spectra and (b) TRPL decay curves of perovskite films with carbon electrode coverage.

**Figure S19**. Statistical PCEs and *J-V* curves of T-2D perovskite tailored CsPbI_2_Br PSCs and LP-2D perovskite tailored CsPbI_2_Br PSCs.

**Figure S20.** *J-V* curves of CsPbI_2_Br PSCs based on control and IPA-treated films.

**Figure S21.** The dependence of *V*_OC_ and *J*_SC_ on light intensity for various devices.

**Figure S22.** Transient photovoltage (TPV) decay curves of various devices.


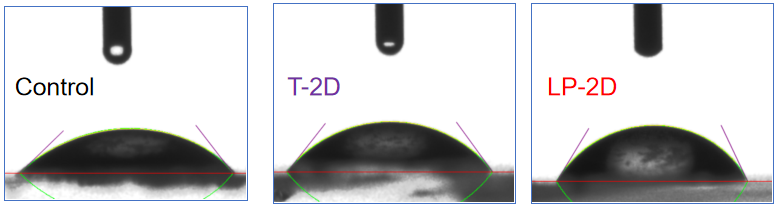


**Figure S23.** Water contact angles on perovskite surface.


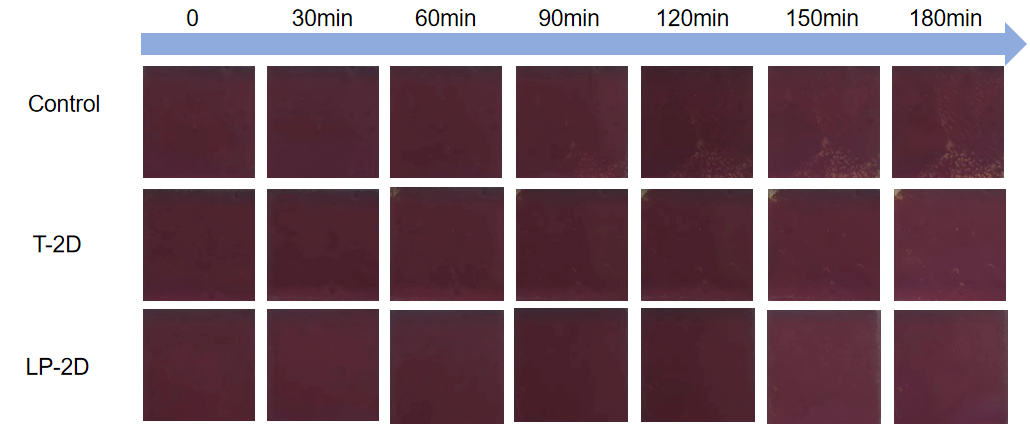


**Figure S24.** Color change of inorganic perovskite films at air condition without any protection.

**Figure S25.** Evolution of UV-vis spectra of CsPbI_2_Br films after exposure under air condition at different times.

**Figure S26.** XRD patterns of CsPbI_2_Br films after exposure under air condition at different times.

**Figure S27.** PL spectra of CsPbI_2_Br films after exposure under air condition at different times.

**Figure S28**. The photovoltaic parameters degradation as a function of storage time under 25 ^o^C, 10% RH.

**Figure S29**. Thermal stability of PSCs stored at 80 ^o^C.

**Table S1.** The photovoltaic parameters of CsPbI_2_Br PSCs based on perovskite films (T-2D) after treatment with various CsBr concentrations under standard illumination.

| Perovskites | c (mg/ml) | *V*_OC_ (V) | *J*_SC_ (mA/cm^2^) | PCE (%) | FF (%) |
| --- | --- | --- | --- | --- | --- |
| CsPbI_2_Br | 0 | 1.245 | 13.77 | 11.80 | 68.95 |
|  | 0.8 | 1.277 | 13.811 | 13.14 | 70.98 |
|  | 1.0 | 1.314 | 14.241 | 14 | 74.83 |
|  | 1.2 | 1.322 | 14.56 | 14.8 | 76.89 |
|  | 1.4 | 1.285 | 14.150 | 13.27 | 72.99 |
|  | 1.6 | 1.313 | 13.998 | 12.97 | 70.56 |

**Table S2**. The photovoltaic parameters of CsPbI_2_Br PSCs based on polished perovskite films (LP-2D) after treatment with various CsBr concentrations under standard illumination.

| Perovskites | c (mg/ml) | *V*_OC_ (V) | *J*_SC_ (mA/cm^2^) | PCE (%) | FF (%) |
| --- | --- | --- | --- | --- | --- |
| CsPbI_2_Br | 0 | 1.245 | 13.77 | 11.80 | 68.95 |
|  | 0.8 | 1.329 | 14.513 | 14.75 | 76.49 |
|  | 1.0 | 1.34 | 14.81 | 15.29 | 77.00 |
|  | 1.2 | 1.322 | 14.428 | 14.31 | 75.05 |
|  | 1.4 | 1.322 | 14.029 | 13.56 | 73.08 |
|  | 1.6 | 1.306 | 14.017 | 13.37 | 73.09 |

# Table S2. Summary of the photovoltaic data for previously reported CsPbI_2_Br (carbon electrode).

| **Based on CsPbI_2_Br (carbon electrode)** | | | | | |
| --- | --- | --- | --- | --- | --- |
| Device architecture | *J*_SC_  (mA cm^−2^) | *V*_OC_  (V) | FF  (%) | PCE  (%) | Ref. |
| FTO/c-TiO_2_/CsPbI_2_Br/(2D/3D)/Carbon | **14.81** | **1.34** | **77** | **15.29** | **This work** |
| FTO/c-TiO_2_/CsPbI_2_Br/Carbon | 14.18 | 1.21 | 75.90 | 13.08 | 1 |
| FTO/TiO_2_/CsPbI_2_Br/Carbon | 15.70 | 1.312 | 74.00 | 15.24 | 2 |
| FTO/TiO_2_/CsPbI_2_Br/NPTMS/Carbon | 14.78 | 1.13 | 61.00 | 10.22 | 3 |
| FTO/c-TiO_2_/CsPbI_2_Br/Carbon | 13.30 | 1.14 | 73.86 | 11.24 | 4 |
| ITO/c-TiO_2_/CsPbI_2_Br/ATHPBr/Carbon | 14.28 | 1.30 | 78.11 | 14.50 | 5 |
| ITO/SnO_2_/SnCl_2_/CsPbI_2_Br/Carbon | 14.74 | 1.26 | 74.00 | 13.78 | 6 |
| ITO/SnO_2_/CsPbI_2_Br/BrAL/Carbon | 13.68 | 1.21 | 68.80 | 11.34 | 7 |
| FTO/TiO_2_/CsPbI_2_Br/Carbon | 14.60 | 1.19 | 73.60 | 12.78 | 8 |
| FTO/TiO_2_/PVP-CsPbI_2_Br/Spiro-OMeTAD/Carbon | 18.47 | 1.01 | 56.35 | 10.47 | 9 |
| FTO/SnO_2_/CsPbI_2_Br/Carbon | 14.51 | 1.30 | 71.02 | 13.38 | 10 |
| FTO/TiO_2_/BMIMBF_6_/CsPbI_2_Br/Carbon | 14.33 | 1.22 | 75.27 | 13.19 | 11 |
| ITO/SnO_2_/CsPbI_2_Br/BMIMBF_4_/Carbon | 14.68 | 1.27 | 75.00 | 14.03 | 12 |
| FTO/TiO_2_/MABr-CsPbI_2_Br/Carbon | 16.62 | 1.21 | 74.00 | 14.84 | 13 |
| FTO/TiO_2_/CsPbI_2_Br/CuPc/Carbon | 14.33 | 1.22 | 75.00 | 13.16 | 14 |
| ITO/SnO_2_/SnCl_2_/CsPbI_2_Br/Cs_2_PtI_6_/Carbon | 14.85 | 1.28 | 72.00 | 13.69 | 15 |
| FTO/c-TiO_2_/m-TiO_2_/CsPbI_2_Br/Carbon black/Carbon | 14.94 | 1.21 | 72.59 | 13.13 | 16 |
| ITO/SnO_2_/CsPbI_2_Br/SnPc/Carbon | 13.69 | 1.24 | 66.90 | 11.39 | 17 |
| FTO/TiO_2_ NRAs/CsPbI_2_Br/Carbon | 14.39 | 1.15 | 69.10 | 11.45 | 18 |
| FTO/TiO_2_/CsPbI_2_Br/Carbon | 14.47 | 1.25 | 80.10 | 14.49 | 19 |
| FTO/c-TiO_2_/m-TiO_2_/Al_2_O_3_/Mg-CsPbI_2_Br/NiO/Carbon | 14.75 | 1.06 | 69.00 | 10.80 | 20 |
| FTO/c-TiO_2_/m-TiO_2_/La-CsPbI_2_Br/Carbon | 11.66 | 1.12 | 61.24 | 8.03 | 21 |
| FTO/SnO_2_/CsPbI_2+x_Br_1-x_/Carbon | 15.46 | 1.23 | 64.00 | 12.19 | 22 |
| ITO/SnO_2_/KOH/CsPbI_2_Br/Carbon | 13.89 | 1.21 | 68.71 | 11.78 | 23 |
| ITO/SnO_2_/CsPbI_2_Br/Co_3_O_4_/Carbon | 13.09 | 1.19 | 72.12 | 11.21 | 24 |
| ITO/SnO_2_/CsPbI_2_Br/PMMA/Carbon | 12.64 | 1.202 | 71.00 | 10.95 | 25 |
| ITO/SnO_2_/CsPbI_2_Br/CuPc/Carbon | 13.61 | 1.22 | 66.30 | 11.04 | 26 |
| ITO/SnO_2_/CsPbI_2_Br/Carbon | 14.25 | 1.14 | 64.12 | 10.44 | 27 |
| FTO/SnO_2_/CsPbI_2_Br/Carbon | 14.29 | 1.33 | 70.96 | 13.52 | 28 |
| ITO/SnO_2_/Nb-CsPbI_2_Br/Carbon | 12.06 | 1.20 | 72.00 | 10.42 | 29 |
| FTO/TiO_2_/CsPbI_2_Br/P3HT-MWCNT/Carbon | 13.35 | 1.21 | 62.00 | 10.01 | 30 |
| FTO/c-TiO_2_/m-TiO_2_/Al_2_O_3_/CsPbI_2_Br/NiO/Carbon | 14.33 | 0.94 | 62.50 | 8.44 | 31 |
| FTO/TiO_2_/CsPbI_2_Br-HTAB/Carbon | 14.10 | 1.26 | 80.60 | 14.30 | 32 |
| ITO/SnO_2_/CsPbI_2_Br/Carbon | 12.91 | 1.19 | 66.10 | 10.13 | 33 |
| FTO/c-TiO_2_/CsPbI_2_Br/Carbon | 13.87 | 1.15 | 64.00 | 10.21 | 34 |
| FTO/c-TiO_2_/CsPbI_2_Br/Carbon | 13.54 | 1.15 | 64.20 | 10.00 | 35 |
| FTO/Nb_2_O_5_/Cs_0.99_Rb_0.01_PbI_2_Br/Carbon | 14.02 | 1.24 | 69.00 | 12.00 | 36 |
| FTO/c-TiO_2_/CsPbI_2_Br/P-QD/Carbon | 14.52 | 1.309 | 79.11 | 15.04 | 37 |
| FTO/SnO_2_-Cl/CsPbI_2_Br-Cl/Carbon | 14.70 | 1.26 | 76.17 | 14.11 | 38 |
| FTO/TiO_2_/CsPbI_2_Br/Carbon | 14.28 | 1.324 | 80.73 | 15.27 | 39 |
| ITO/SnO_2_/KTFA/CsPbI_2_Br/CF_3_PMABr/Carbon | 14.98 | 1.273 | 73.7 | 14.05 | 40 |
| ITO/SnO_2_/MAAc-CsPbI_2_Br/Carbon | 14.20 | 1.27 | 61.80 | 11.20 | 41 |
| ITO/c-TiO_2_/CsPbI_2_Br/Carbon | 14.21 | 1.28 | 79.40 | 14.46 | 42 |
| ITO/SnO_2_/SnCl_2_/CsPbI_2_Br/PTU/Carbon | 14.83 | 1.22 | 72.00 | 13.01 | 43 |
| ITO/SnO_2_/ZnO/E-K/CsPbI_2_Br/Carbon | 14.95 | 1.255 | 74.30 | 13.94 | 44 |
| FTO/SnO_2_/CsPbI_2_Br+3Br-PABr/Carbon | 14.60 | 1.28 | 75.14 | 14.04 | 45 |
| ITO/SnO_2_/ZnO-EAD/CsPbI_2_Br/Carbon | 14.98 | 1.272 | 76.50 | 14.58 | 46 |
| FTO/c-TiO_2_/m-TiO_2_/CsPb_2_I_4_Br-CsPbI_2_Br BHJ/Carbon | 14.60 | 1.32 | 79.11 | 15.25 | 47 |
| ITO/SnO_2_/ZnO/CsPbI_2_Br/P-F-PEABr/Carbon | 14.91 | 1.269 | 73.80 | 13.97 | 48 |
| FTO/TiO_2_@Sb_2_S_3_-MPA/CsPbI_2_Br/Carbon | 14.50 | 1.29 | 77.49 | 14.59 | 49 |
| FTO/SnO_2_/CsPbI_2_Br/Fe_3_O_4_@NC UVO/Carbon | 15.31 | 1.26 | 64.00 | 12.25 | 50 |
| FTO/SnO_2_/Pb(Ac)_2_-CsPbI_2_Br/Carbon | 15.87 | 1.32 | 65.00 | 13.73 | 51 |
| FTO/TiO_2_/CsPbI_2_Br/CNT/P3HT/Carbon | 14.63 | 1.355 | 78.50 | 15.56 | 52 |
| FTO/SnO_2_/AA-CsPbI_2_Br/Carbon | 14.50 | 1.22 | 72.00 | 12.71 | 53 |
| FTO/TiO_2_/NaSCN-CsPbI_2_Br/Carbon | 14.31 | 1.267 | 80.70 | 14.63 | 54 |
| FTO/SnO_2_/CdCl_2_/CsPbI_2_Br/Carbon | 14.30 | 1.30 | 77.85 | 14.47 | 55 |
| FTO/TiO_2_+MXene/CsPbI_2_Br/Carbon | 14.961 | 1.289 | 80.26 | 15.48 | 56 |
| FTO/TiO_2_/CsPbI_2_Br/EAI+PEAI/Carbon | 14.59 | 1.17 | 80.32 | 13.76 | 57 |
| FTO/TiO_2_/CsPbI_2_Br/CSI/Carbon | 14.51 | 1.267 | 79.81 | 14.67 | 58 |
| FTO/c-TiO_2_/CsPbI_2_Br/NiOx/NiOx@Carbon | 14.82 | 1.31 | 77.31 | 15.01 | 59 |
| FTO/c-TiO_2_/CsPbI_2_Br-MWCNT/Carbon | 14.85 | 1.291 | 79.00 | 15.14 | 60 |
| ITO/SnO_2_/CsPbI_2_Br/1-B-3-MTMPF_6_/Carbon | 14.69 | 1.200 | 76.00 | 13.47 | 61 |
| FTO/TiO_2_/ET/CsPbI_2_Br/Carbon | 14.14 | 1.308 | 78.71 | 14.56 | 62 |
| FTO/TiO_2_/PFPA/CsPbI_2_Br/Carbon | 14.79 | 1.21 | 79.07 | 14.15 | 63 |
| ITO/SnO_2_/CsPbI_2_Br/BMIMMS/Carbon | 15.13 | 1.23 | 71.2 | 13.25 | 64 |
| FTO/SnO_2_/CsPbI_2_Br/DC/Carbon | 15.96 | 1.26 | 70 | 14.08 | 65 |
| FTO/TiO_2_-BAP/CsPbI_2_Br/PCBM/Carbon | 14.88 | 1.27 | 78.99 | 14.9 | 66 |
| FTO/TiO_2_/CsPbI_2_Br-PFPA/Carbon | 14.8 | 1.21 | 79.07 | 14.15 | 67 |
| FTO/TiO_2_/CsPbI_2_Br/(Sb_2_S_3_/PbS)/Carbon | 14.93 | 1.28 | 77.11 | 14.73 | 68 |
| FTO/TiO_2_/CsPbI_2_Br/(0D/3D)/Carbon | 15.91 | 1.33 | 72 | 15.24 | 69 |
| FTO/SnO_2_/CsPbI_2_Br-Ln(OTF)_3_/Tm(OTF)_3_/Carbon | 14.96 | 1.312 | 77.1 | 15.13 | 70 |
| FTO/SnO_2_/CsPbI_2_Br/F_3_EACl/Carbon | 14.6 | 1.28 | 78.62 | 14.69 | 71 |
| FTO/SnO_2_-PS/CsPbI_2_Br/Carbon | 14.12 | 1.25 | 74.5 | 13.11 | 72 |
| FTO/c-TiO_2_/CsPbI_2_Br-TRP/Carbon | 14.76 | 1.30 | 80.85 | 15.51 | 73 |
| FTO/c-TiO_2_/CsPbI_2_Br /Carbon | 14.4 | 1.344 | 79.43 | 15.37 | 74 |
| FTO/c-TiO_2_/IMA/CsPbI_2_Br-BTFBT/Carbon | 14.576 | 1.322 | 78.59 | 15.12 | 75 |

**References**

1. K. Zhang, W. Li, J. Yu, X. Han, *Sol. Energy* **2021**, *222*, 186–192.
2. W. Zhu, J. Ma, W. Chai, T. Han, D. Chen, X. Xie, G. Liu, P. Dong, H. Xi, D. Chen, *Sol. RRL* **2022**, *6*, 2200020.
3. J. Yu, W. Li, K. Zhang, X. Han, *J. Mater. Sci. Mater. Electron.* **2021**, *32*, 20936–20945.
4. W. Su, X. Han, J. Feng, Z. Zhu, H. Huang, J. Li, T. Yu, Z. Li, Z. Zou, *Energ. Fuel.* **2021**, *35*, 11488–11495.
5. Z. Yan, D. Wang, Y. Jing, X. Wang, H. Zhang, X. Liu, S. Wang, C. Wang, W. Sun, J. Wu, *Chem. Eng. J.* **2022**, 433, 134611.
6. Q. Han, S. Yang, L. Wang, F. Yu, C. Zhang, M. Wu, T. Ma, *Sol. Energy* **2021**, *216*, 351–357.
7. Y. Li, J. Yang, L. Xie, Y. Li, X. Liang, X. Lu, X. Gao, J. Gao, L. Shui, S. Wu, J.-M. Liu, *ACS Appl. Energy Mater.* **2021**, *4*, 5415–5423.
8. K. Wang, T. You, R. Yin, B. Fan, J. Liu, S. Cui, H. Chen, P. Yin, *ACS Appl. Energy Mater.* **2021**, *4*, 3508–3517.
9. S. Ullah, P. Yang, J. Wang, L. Liu, S.-E. Yang, T. Xia, Y. Chen, *J. Solid State. Chem.* **2022**, *305*, 122656.
10. Y. Wu, Q. Zhang, L. Fan, C. Liu, M. Wu, D. Wang, T. Zhang, *ACS Appl. Energy Mater.* **2021**, *4*, 5583–5589.
11. R. Yin, K.-X. Wang, S. Cui, B.-B. Fan, J.-W. Liu, Y.-K. Gao, T.-T. You, P.-G. Yin, *ACS Appl. Energy Mater.* **2021**, *4*, 9294–9303.
12. F. Yu, Q. Han, L. Wang, S. Yang, X. Cai, C. Zhang, T. Ma, *Sol. RRL* **2021**, *5,* 2100404.
13. W. Zhu, W. Chai, D. Chen, J. Ma, D. Chen, H. Xi, J. Zhang, C. Zhang, Y. Hao, *ACS Energy Lett.* **2021**, *6*, 1500–1510.
14. P. Xie, G. Zhang, Z. Yang, Z. Pan, Y. Fang, H. Rao, X. Zhong, *Sol. RRL* **2020**, *4*, 2000431.
15. Q. Han, S. Yang, L. Wang, F. Yu, X. Cai, T. Ma, *J. Colloid Interf. Sci.* **2022**, *606*, 800–807.
16. S. Gong, H. Li, Z. Chen, C. Shou, M. Huang, S. Yang, *ACS Appl. Mater. Inter.* **2020**, *12*, 34882–34889.
17. X. Zhang, N. Gao, Y. Li, L. Xie, X. Yu, X. Lu, X. Gao, J. Gao, L. Shui, S. Wu, J.-M. Liu, *ACS Appl. Energy Mater.* **2020**, *3*, 7832–7843.
18. W. Cai, Y. Lv, K. Chen, Z. Zhang, Y. Jin, X. Zhou, *Energ. Fuel.* **2020**, *34*, 11670–11678.
19. S. Xu, C. Kang, Z. Huang, Z. Zhang, H. Rao, Z. Pan, X. Zhong, *Sol. RRL* **2022**, *6*, 2100989.
20. S. Liu, L. Guan, T. Zhang, X. Gong, X. Zhao, Q. Sun, X. Shai, X. L. Zhang, X. Xiao, Y. Shen, M. Wang, *Appl. Mater. Today* **2020**, *20*, 100644.
21. S. Chen, T. Zhang, X. Liu, J. Qiao, L. Peng, J. Wang, Y. Liu, T. Yang, J. Lin, *J. Mater. Chem. C* **2020**, *8*, 3351–3358.
22. C. Liu, M. Wu, Y. Wu, D. Wang, T. Zhang, *J. Power Sources* **2020**, *447*, 227389.
23. F. Deng, X. Li, X. Lv, J. Zhou, Y. Chen, X. Sun, Y.-Z. Zheng, X. Tao, J.-F. Chen, *ACS Appl. Energy Mater.* **2020**, *3*, 401–410.
24. Y. Zhou, X. Zhang, X. Lu, X. Gao, J. Gao, L. Shui, S. Wu, J.-M. Liu, Sol. RRL **2019**, 3, 1800315.
25. X. Zhang, Y. Zhou, Y. Li, J. Sun, X. Lu, X. Gao, J. Gao, L. Shui, S. Wu, J.-M. Liu, J. Mater. Chem. C **2019**, 7, 3852–3861.
26. X. Zhang, J. Yang, L. Xie, X. Lu, X. Gao, J. Gao, L. Shui, S. Wu, J.-M. Liu, Dyes Pigments **2021**, 186, 109024.
27. Z. Ye, J. Zhou, J. Hou, F. Deng, Y.-Z. Zheng, X. Tao, *Sol. RRL* **2019**, *3,* 1900109.
28. C. Liu, J. He, M. Wu, Y. Wu, P. Du, L. Fan, Q. Zhang, D. Wang, T. Zhang, *Sol. RRL* **2020**, *4*, 2000016.
29. Z. Guo, S. Zhao, A. Liu, Y. Kamata, S. Teo, S. Yang, Z. Xu, S. Hayase, T. Ma, *ACS Appl. Mater. Inter.* **2019**, *11*, 19994–20003.
30. G. Wang, J. Liu, K. Chen, R. Pathak, A. Gurung, Q. Qiao, *J. Colloid Interface Sci.* **2019**, *555*, 180–186.
31. T. Zhang, H. Li, S. Liu, X. Wang, X. Gong, Q. Sun, Y. Shen, M. Wang, *J. Phys. Chem. Lett.* **2019**, *10*, 200–205.
32. G. Zhang, P. Xie, Z. Huang, Z. Yang, Z. Pan, Y. Fang, H. Rao, X. Zhong, *Adv. Funct. Mater.* **2021**, *31*, 2011187.
33. X. Meng, Z. Wang, W. Qian, Z. Zhu, T. Zhang, Y. Bai, C. Hu, S. Xiao, Y. Yang, S. Yang, *J. Phys. Chem. Lett.* **2019**, *10*, 194–199.
34. C. Dong, X. Han, W. Li, Q. Qiu, J. Wang, *Nano Energy* **2019**, *59*, 553–559.
35. C. Dong, X. Han, Y. Zhao, J. Li, L. Chang, W. Zhao, *Sol. RRL* **2018**, *2,* 1800139.
36. Y. Guo, F. Zhao, J. Tao, J. Jiang, J. Zhang, J. Yang, Z. Hu, J. Chu, *ChemSusChem* **2019**, *12*, 983–989.
37. J. Zhang, G. Zhang, Y. Liao, Z. Pan, H. Rao, X. Zhong, *Chem. Eng. J.* **2023**, 453, 139842.
38. K. Wang, W. Sun, W. Liu, X. Huo, R. Yin, J. Liu, Y. Gao, T. You, P. Yin, *Chem. Eng. J*. **2022**, *445*, 136781.
39. G. Zhang, J. Zhang, Z. Yang, Z. Pan, H. Rao, X. Zhong, *Adv. Mater*. **2022**, *34*, 2206222.
40. X. Zhang, D. Zhang, Y. Zhou, Y. Du, J. Jin, Z. Zhu, Z. Wang, X. Cui, J. Li, S. Wu, *Adv. Funct. Mater*. **2022**, *32*, 2205478.
41. X. Li, Y. Zhang, G. Liu, Z. Zhang, L. Xiao, Z. Chen, B. Qu, *ACS Appl. Energy Mater.* **2021**, *4*, 13444−13449.
42. W. Wang, Y. Lin, G. Zhang, C. Kang, Z. Pan, X. Zhong, H. Rao, *J. Energy Chem.* **2021**, *63*, 442–451.
43. Q. Han, F. Yu, L. Wang, S. Yang, X. Cai, X. Meng, O. Yuta, K. Takeshi, C. Zhang, T. Ma, *J. Power Sources* **2021**, *516*, 230676.
44. D. Zhang, X. Zhang, T. Guo, J. Zou, Y. Zhou, J. Jin, Z. Zhu, Q. Cao, J. Zhang, Q. Tai, *Small* **2023**, 19, 2205604.
45. X. Huo, W. Sun, K. Wang, W. Liu, R. Yin, Y. Sun, Y. Gao, T. You, P. Yin, *ACS Appl. Mater. Inter.* **2023**, 15, 9382−9391.
46. D. Zhang, X. Zhang, T. Guo, J. Jin, J. Zou, Z. Zhu, Y. Zhou, Q. Cao, J. Zhang, Z. Ren, Q. Tai, *ACS Appl. Mater. Inter.* **2023**, 15, 10897−10906.
47. C. Kang, S. Xu, H. Rao, Z. Pan, X. Zhong, *ACS Energy Lett.* **2023**, 8, 909−916.
48. X. Zhang, D. Zhang, T. Guo, C. Zheng, Y. Zhou, J. Jin, Z. Zhu, Z. Wang, X. Cui, S. Wu, J. Zhang, Q. Tai, *J. Mater. Chem. C* **2022**, *10*, 15573–15581.
49. Y. Jing, X. Liu, Y. Xu, M. Zhang, R. Li, S. Wang, Z. Yan, W. Sun, J. Wu, Z. Lan, *Chem. Eng. J.* **2023**, 455, 140871.
50. X. Yang, Y. Qi, P. Wei, Q. Hu, J. Cheng, Y. Xie, *J. Power Sources* **2023**, 566, 232927.
51. S. Liu, X. Xu, C. Xing, G. Ge, D. Wang, T. Zhang, *Energy Technol.* **2022**, *10*, 2200378.
52. G. Zhang, J. Zhang, Z. Pan, H. Rao, X, Zhong, *Sci. China Mater.* **2023**, 66, 1727–1735.
53. S. Zheng, H. Wang, J. Li, P. Wei, Y. Qi, Y. Xie, *Opt. Mater.* **2023**, 136, 113427.
54. Z. Yang, G. Zhang, J. Zhang, Z. Pan, S. Yan, B. Liu, H. Rao, X. Zhong, *Chem. Eng. J.* **2022**, *430*, 133083.
55. X. Qiu, Y. Xu, R. Li, Y. Jing, Z. Yan, F. Liu, L. Wu, Y. Tu, J. Shi, Z. Du, J. Wu, Z. Lan, *Small* **2023**, 19, 2206245.
56. Y. Xu, F. Liu, R. Li, Y. Jing, Q. Chen, X. Chen, C. Deng, Z. Du, W. Sun, J. Wu, Z. Lan, *Chem. Eng. J.* **2023**, 461, 141895.
57. X. Huo, K. Wang, R. Yin, W. Sun, Y. Sun, Y. Gao, T. You, P. Yin, *Sol. Energ. Mat. Sol. C* **2022**, *247*, 111963.
58. G. Zhang, J. Zhang, Y. Liao, Z. Pa, H. Rao, X. Zhong, *Chem. Eng. J.* **2022**, *440*, 135710.
59. B. Xu, D. Liu, C. Dong, M. Awais, W. Wang, Y. Song, Y. Deng, M. Yao, J. Tong, G. Yue, W. Zhang, F. Tan, M. I. Saidaminov, *J. Colloid Interface Sci.* **2023**, 641, 105–112
60. J. Li, J. Duan, Q. Guo, Z. Qi, X.Duan. *Adv. Funct. Mater*. **2023**, 33, 2308036.
61. W. Zhan, L. Wu. J. Chen, J. Ju, Y. Zeng, *ChemSusChem*. **2024**, DOI: https://doi.org/10.1002/cssc.202400223.
62. Z. Qi, J.Li, X. Zhang, J. Dou, Q.Guo, *ACS Appl. Mater. Inter.* **2024**, 16, 14974-83.
63. X. Huo, Y. Jiang, J. Lv, W. Sun, R. Yin, *Chem. Eng. J.* **2024**, 484, 149626.
64. W. Ye, Y. Zeng, J. Chen, J. He, Y. Zou, R. Yang, J. Huang,Z. Peng, J. Chen, *Mater. Today Energy*, **2025**, 48, 101799.
65. M. He, C. Xing, Q. Bao, L. Yu, Z. Nie, R. Wang, C. Wan, D. Wang, T. Zhang, *ACS Appl. Mater. Inter.* **2024**, 16, 62392−62401.
66. T. Yun, H. Cai, W. Lyu, X. Lu, X. Gao, J. Liu, S. Wu, *ACS Appl. Mater. Inter.* **2024**, 16, 57412−57420.
67. X. Huo, Y. Jiang, J. Lv, W. Sun, W. Liu, R. Yin, Y. Gao, K. Wang, T. You, P. Yin, *Chem. Eng. J*. **2024**, 484,149626.
68. F. Liu, H. Deng, Y. Jing, L. Gao, Y. Xu, R. Li, C. Deng, D. Wang, Z. Du, L. Yang, Y. Cao, J. Wu, Z. Lan, *Chem. Eng. J*. **2025**, 508,161029.
69. D. Wang, C. Song, L. Cheng, K. Chen, F. Meng, G. Wanga, W. Xiang, *Chem. Eng. J*. **2025**, 512, 162516.
70. W. Li, H. Tong, Y. Li, X. Liu, G. Wan, X. Ma, H. Liu, Z. Gao, Y. Fu, D. He, Z. Li, J. Li, *Small* **2024**, 20, 2406784.
71. F. Liu, Y. Xu, R. Li, C. Deng, Li. Gao, H. Deng, Y. Jing, D. Jiang, Q. Yao, C. Zhong, D. Wang, J. Wu, Z. Lan, *Chem. Eng. J*. **2024**, 500, 157370.
72. Y. Shi, L. Zhang, S. Hu, X. Wang, J. Han, J. Huang, J. Chen, Y. Zhang, X. Zhang, J. He, H. Zuo, J. Ju, Z. Wu, W. Zhao, Y. Zeng, Y. Zou, K. Liao, R. Yang, W. Ye, Y. Gu, L. Gong, S. Fan, Z. Peng, J. Chen, *Chem. Eng. J.* **2024,** 492, 152210.
73. S. Geng, J. Duan, N. Liu, H. Li, X. Zhu, X. Duan, Q. Guo, J. Dou, B. He, Y. Zhao, Q. Tang, *Angew. Chem. Int. Ed*. **2024**, 63, e202407383.
74. H. Li, J. Duan, C. Zhang, N. Liu, L. Ma, X. Duan, J. Dou, Q. Guo, B. He, Y. Zhao, Q. Tang, *Angew. Chem. Int. Ed*. **2024**, e202419061.
75. N. Liu, J. Duan, C. Zhang, J. Zhang, Y. Bi, L. Ma, D. Xu, J. Gao, X. Duan, J. Dou, Q. Guo, B. He, Y. Zhao, Q. Tang, *Angew. Chem. Int. Ed.* **2025**, 64, e202424046.
